# Estimation of skeletal kinematics in freely moving rodents

**DOI:** 10.1038/s41592-022-01634-9

**Published:** 2022-10-17

**Authors:** Arne Monsees, Kay-Michael Voit, Damian J. Wallace, Juergen Sawinski, Edyta Charyasz, Klaus Scheffler, Jakob H. Macke, Jason N. D. Kerr

**Affiliations:** 1 Department of Behavior and Brain Organization, Max Planck Institute for Neurobiology of Behavior, Bonn, Germany; 2 High-Field MR Center, Max Planck Institute for Biological Cybernetics, Tübingen, Germany; 3 Department for Biomedical Magnetic Resonance, Eberhard Karls University of Tübingen, Tübingen, Germany; 4 Machine Learning in Science, Eberhard Karls University of Tübingen, Tübingen, Germany; 5 Empirical Inference, Max Planck Institute for Intelligent Systems, Tübingen, Germany

**Keywords:** Software, Computational neuroscience, Rat, Mouse, Motor control

## Abstract

Forming a complete picture of the relationship between neural activity and skeletal kinematics requires quantification of skeletal joint biomechanics during free behavior; however, without detailed knowledge of the underlying skeletal motion, inferring limb kinematics using surface-tracking approaches is difficult, especially for animals where the relationship between the surface and underlying skeleton changes during motion. Here we developed a videography-based method enabling detailed three-dimensional kinematic quantification of an anatomically defined skeleton in untethered freely behaving rats and mice. This skeleton-based model was constrained using anatomical principles and joint motion limits and provided skeletal pose estimates for a range of body sizes, even when limbs were occluded. Model-inferred limb positions and joint kinematics during gait and gap-crossing behaviors were verified by direct measurement of either limb placement or limb kinematics using inertial measurement units. Together we show that complex decision-making behaviors can be accurately reconstructed at the level of skeletal kinematics using our anatomically constrained model.

## Abstract (web summary)

Pose estimation in combination with an anatomically constrained model allows inferring skeletal kinematics in rodents.

## Main

Much of the motion kinematic data forming our view of the sensorimotor control of movement was collected during short behavioral epochs where the animal was in various forms of restraint^1–6^, but a major challenge still remains for generating detailed kinematics of individual body parts, such as limbs, and how they interact with the environment during free behavior^7,8^. This poses an especially difficult problem as limb motions involving muscles, bones and joints are biomechanically complex given their three-dimensional (3D) translational and rotational co-dependencies^9,10^. Single-plane X-ray-based cineradiography and fluoroscopy approaches can be used to directly image bone motion during gait^11,12^ for calculation of limb kinematics, but are limited in simultaneous field of view^13^, temporal sample rate^14,15^ (but see elsewhere^16^) and can only image one plane. A combination of multiple X-ray sources^17,18^ and 3D modeled bones^19^ have been used to measure single-limb joint kinematics in multiple rotational planes, from animals of a variety of different species, while they were walking^20–22^ or performing reaching tasks^23,24^; however, in these experiments the animal’s range of movements was limited to the area illuminated by the X-ray sources.

More recently, imaging of free animal behavior using light^25,26^ has been combined with machine-learning approaches to enable limb tracking in freely moving^27^ and head-restrained insects^28^, and body tracking in multiple species, including humans^29^. While the insect exoskeleton provides joint angle limits and hard limits of limb position and can be tracked as a surface feature, when imaging vertebrates such as rodents, fur and soft tissue occludes the entire skeleton, complicating inference of bone positions, as the spatial relationship between skeleton and overlying soft tissues are less apparent^12,30–32^. Despite this limitation, recent approaches have extended two-dimensional surface-tracking methods^27,33,34^ to include 3D pose reconstructions^35,36^ using a multi-camera cross-validation approach and hand-marked ground-truth datasets^37^, allowing general kinematic representation of animal behaviors and poses for multiple species^29,38^, as well as simultaneous measurements from multiple animals^27,33,39^. Extending these approaches to obtain skeletal kinematics relies on knowledge of the skeletal anatomy and biomechanics as well as motion restrictions of joints^9^, because animal poses are limited by both bone lengths and joint angle limits.

Here, we developed an anatomically constrained skeleton model incorporating mechanistic knowledge of bone locations, anatomical limits of bone rotations and temporal constraints, to track 3D joint positions and their kinematics in freely moving rats and mice. We compared the performance of our approach with ground-truth data, using magnetic resonance imaging (MRI) for comparison with the initial skeleton fitting, frustrated internal reflection for comparison of foot-placement positions with positions inferred from our method and direct measurements of limb kinematics using inertial measurement units (IMUs) for comparison with the inferred skeletal kinematics. Together the fully constrained skeleton enabled the reconstruction of skeleton poses and kinematic quantification during gap-crossing, jumping and reaching tasks and throughout spontaneous behavioral sequences.

## Results

**Fig. 1:**
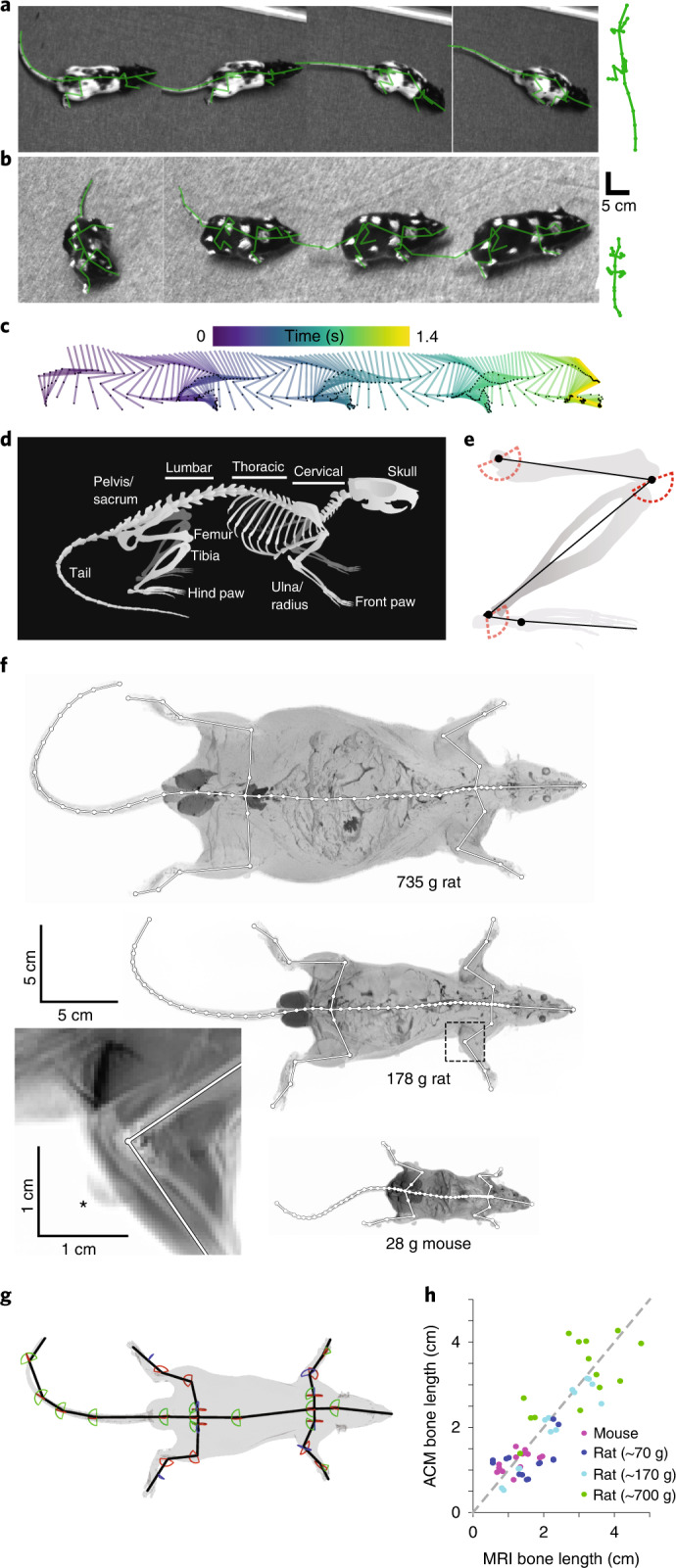
Learning an anatomically constrained skeleton model for mice and rats. **a**, Example images of a freely moving rat with painted surface labels, also showing the fitted and re-projected skeleton model (green). Scaled skeleton shown at right for comparison with **b**. **b**, as for **a**, but showing images from a freely moving mouse. Scaled skeleton on right for comparison with **a**. **c**, Time series of the reconstructed right hind limb during the sequence shown in **a**. **d**, Schematic image of a rat skeleton showing anatomical landmarks. **e**, Schematic image of a hind limb with modeled bones (black lines) and joints (black dots) as well as enforced joint angle limits for flexion and extension (red dashed lines). **f**, MRI scans (maximum projection) of two rats of different weights (top, middle), a mouse (bottom) and an enlargement of the right elbow joint from a rat (bottom left, mean projection, area denoted by dashed box) with manually labeled bone (white lines) and joint (white dots) positions. Note visible MRI surface marker (asterisk). **g**, 3D representation of a rat’s MRI scan showing the animal’s surface (gray) and the aligned skeleton model (black lines) and joint angle limits for flexion or extension (red lines), abduction or adduction (green lines) and internal or external rotation (blue lines). **h**, Learned bone lengths compared to MRI bone lengths (*n* = 6 rats and 2 mice). Colors represent mouse data (magenta) and small (blue, 71 g and 72 g), medium (cyan, 174 g and 178 g) and large (green, 699 g and 735 g) rat data.

We tracked 3D skeletal joint positions and their kinematics in freely moving rats (Fig. 1a; *n* = 8; average weight 284.4 g; range 71–735 g) and mice (Fig. 1b bottom; *n* = 2; average weight 32.8 g; range 27–36 g) using videography and an anatomically constrained skeleton model (ACM) incorporating mechanistic knowledge of bone locations, anatomical limits of bone rotations, and temporal constraints. We performed pose and kinematic estimation using the ACM in three steps: (1) a manual-labeling initialization step, (2) a surface-marker-detection step and (3) a pose-estimation step from which kinematics of individual joints could be calculated. In the first step, we manually labeled the surface markers in a subset of images throughout the dataset. These data were used both for training the DeepLabCut^34^ (DLC) network and for learning the model skeleton in the pose-estimation step. In the second step, we used DLC to automatically detect surface-marker positions located on the behaving animals. In the third step, the model skeleton was first learned from a subset of images and then pose estimates were made for each frame using the learned skeleton and the expectation-maximization (EM) algorithm. From this last step, the kinematics could be calculated for each joint (Fig. 1c). These steps are described in further detail below.

### Building and constraining the skeleton model

At the core of this approach was a generalized skeleton, based on both rat^40^ and mouse bone anatomy^41,42^ (Fig. 1d), modeled as a mathematical graph with vertices representing individual joints and edges representing bones (Fig. 1e, Supplementary Fig. 1a and Supplementary Text). For example, we used a single edge to represent the animal’s head, we approximated the spinal column using four edges based on cervical, thoracic and lumbar sections of the column with the sacrum as the fourth edge^40–42^ and we approximated the tail using five edges (Fig. 1d and Supplementary Fig. 1).

To constrain the skeleton model we applied angle limits for each joint based on measured rotations^43^ (Fig. 1e) as well as anatomical constraints based on measured relationships between bone lengths and animal weight for rats^44^, and on measured adult bone lengths for mice^42^. Finally, as vertebrates are symmetrical around the mid-sagittal plane we applied an additional anatomical constraint to ensure symmetry for bone lengths and surface-marker locations (Supplementary Fig. 1). Together, this approach enabled fitting of a skeleton model for each animal. To generate probabilistic estimates of 3D joint positions and provide temporal constraints, we implemented a temporal unscented Rauch–Tung–Striebel (RTS) smoother^45,46^, an extension of the Kalman filter^47^, which is suitable for nonlinear dynamic models and also incorporates information from future marker locations (Supplementary Text). Parameters of the smoother were learned via the EM algorithm^48^, by iteratively fitting poses of the entire behavioral sequence.

### Learning the skeleton for individual animals

To relate the animal’s surface to the underlying skeleton we used a grid of rationally placed surface markers on each animal, which were either distinct anatomical landmarks such as the snout or were painted on the animal’s fur (Fig. 1a (14 landmarks and 29 spots in total per animal) and Supplementary Fig. 1). In the model each marker was rigidly connected to at least a single joint, but one joint could be associated with multiple markers, making the fitting of the model skeleton more robust to variation in the number of visible markers and surface-marker position relative to the joint during animal movement. We then imaged each marked animal using overhead cameras as it freely behaved, and we manually annotated the visible surface markers from each camera from a fraction of all recorded images to tailor the generalized skeleton model to the individual animal (Supplementary Fig. 2). For this, we utilized a gradient descent approach, minimizing the 2D distance between manual labels and projected 3D positions into each camera. We simultaneously optimized all per-frame pose parameters (position and bone rotation) and the skeletal parameters (bone length and relative position of markers to joints), which remain constant over time and define the final individual skeleton for the subsequent pose estimation of the animal (Supplementary Video 1). To evaluate the accuracy of the skeleton model we generated high-resolution MRI scans for each rat and mouse (Fig. 1f; *n* = 6 rats, *n* = 2 mice) and aligned the skeleton model to measured positions of 3D surface markers (Fig. 1g). Inferred bone lengths were not significantly different from those measured in MRI scans (Fig. 1h; mouse and rat bone length error of 0.45 ± 0.35 and 0.36 cm (mean ± s.d. and median); *n* = 56 bone lengths; Spearman correlation coefficient of 0.81; two-tailed *P* value testing non-correlation of 2.86 × 10^−14^; range of measured bone lengths 0.56–4.76 cm). Together this demonstrated that the ACM generated by our algorithm was accurate when compared to the animal’s actual skeleton across the range of animal sizes and species.

### Accurate behavior reconstructions required both temporal and anatomical constraints

**Fig. 2:**
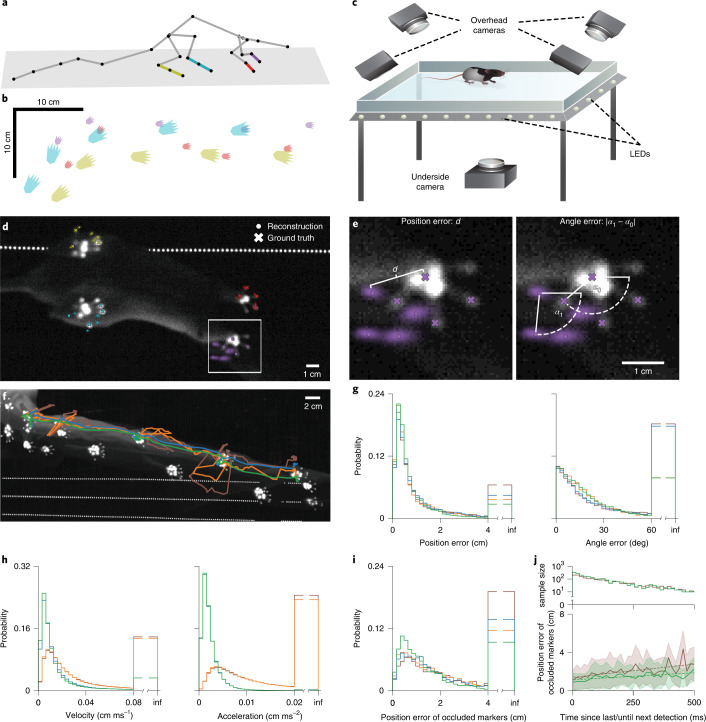
Comparison between inferred and measured paw positions during free behavior. **a**, Reconstructed animal pose based on a learned skeleton model with highlighted left front (purple), right front (red), left hind (cyan) and right hind paw (yellow). **b**, Reconstructed *x–y* positions of the paws during gait. Colors as in **a**. **c**, Schematic image of the FTIR touch-sensing setup with one underneath and four overhead cameras. **d**, Single image from the underneath camera with reconstructed (*x*) and ground-truth (filled circle) *x–y* positions of the paw’s centers and fingers/toes for all four paws. Colors as in **a**. Large point clouds around landmark locations indicate high uncertainty. Note that only the second toe and finger are represented in the model skeleton, but that the positions of three toes and fingers were detected and tracked. **e**, Enlarged view of the left front paw in **d** (white box) showing calculation of position error (left) and the angle error (right). Scale bar in right image applies to both images in **e**. **f**, Maximum intensity projection from the underneath camera of a 2.5-s long sequence with trajectories for the reconstructed *x–y* positions of the right hind paw using the ACM (green), temporal (blue), joint angle (orange) and naive skeleton (brown) models. **g**, Probability histograms for paw position (left) and angle errors (right) comparing different model constraint regimes. Color-coding as in **f**. **h**, Probability histograms for paw velocities (left) and accelerations (right) comparing different model constraint regimes. Color-coding as in **f**. **i**, Probability histograms for paw position errors when only undetected surface markers are used for the calculation comparing different model constraint regimes. Color-coding as in **f**. **j**, Position errors of occluded markers (bottom, mean ± s.d. of samples) and corresponding binned sample sizes (top) as a function of time since last or until next marker detection comparing different model constraint regimes. Color-coding as in **f**. Sample sizes differ depending on whether reconstructed poses were obtained via the unscented RTS smoother (green) or not (brown).

To reconstruct behavioral sequences using the ACM, we first tracked two-dimensional (2D) surface-marker locations in the recorded movies using DLC trained with the manually marked frames. As the ACM contained both joint angle limits and temporal constraints, we evaluated the role of these by reconstructing poses without either the joint angle limits or the temporal constraints. We compared the resulting temporal model, joint angle model and naive skeleton model, constrained by neither, to the ACM. Freely behaving animals showed many spontaneous behaviors, such as rearing and gait (Supplementary Video 2). We used a modified frustrated total internal reflection (FTIR) touch-sensing approach^49,50^ (Fig. 2a–c and Supplementary Video 3) to generate ground-truth animal paw positions and orientations during gait, and then compared these measurements to the paw positions and orientations inferred by each model variation (Fig. 2d–f; *n* = 6 animals; 29 sequences; and 181.25 s per 145,000 frames in total from four cameras). The ACM produced significantly smaller positional errors compared to all other models (Fig. 2g; 10,410 positions in total; *P* values using one-sided Kolmogorov–Smirnov test, *P* = 9.84 × 10^−21^ for ACM versus joint angle model; *P* = 4.38 × 10^−35^ for ACM versus temporal model; and *P* = 9.03 × 10^−37^ for ACM versus naive skeleton model), whereas orientation errors were only significantly smaller when comparing the ACM to the temporal and naive skeleton model (Fig. 2g; 7,203 and 6,969 orientations in total for the ACM and joint angle model and the temporal and naive skeleton model, respectively; *P* values using one-sided Kolmogorov–Smirnov test, *P* = 3.20 × 10^−39^ for ACM versus temporal model; and *P* = 2.51 × 10^−50^ for ACM versus naive skeleton model). While orientation errors were substantially reduced by the anatomical constraints, including temporal constraints limited abrupt pose changes over time compared to either the naive skeleton model or joint angle model (Fig. 2f and Supplementary Videos 4–9). As a result, ACM-generated joint velocities and accelerations (Fig. 2h; 576,288 velocities and accelerations total) were significantly smaller when compared to the joint angle and naive skeleton models (*P* values using one-sided Kolmogorov–Smirnov test were all numerically 0 for ACM versus joint angle model (velocity); ACM versus naive skeleton model (velocity); ACM versus joint angle model (acceleration); and ACM versus naive skeleton model (acceleration)). The temporal and anatomical constraints each had an advantage over the naive skeleton model and both constraints applied simultaneously improved positional accuracy as well as motion trajectories and prevented anatomically infeasible bone orientations and abrupt paw relocations. Moreover, the fraction of position errors exceeding 4 cm increased when constraints were not considered (ACM, 2.72%; joint angle model, 3.64%; temporal model, 4.42%; and naive skeleton model, 6.44%) and the same was observed for orientation errors exceeding 60° (ACM, 7.78%; joint angle model, 7.81%; temporal model, 17.77%; and naive skeleton model, 18.22%). Likewise, enforcing constraints also lowered the percentage of velocities exceeding 0.08 cm ms^−1^ (ACM, 3.29%; joint angle model, 13.49%; temporal model, 3.28%; and naive skeleton model, 13.85%) and accelerations exceeding 0.02 cm ms^−2^ (ACM, 0.22%; joint angle model, 23.43%; temporal model, 0.25%; and naive skeleton model, 24.55%). The ACM was robust to missing surface markers (in cases where surface markers were undetected; Fig. 2i; 2,797 position errors in total) and produced significantly lower errors (*P* values using one-sided Kolmogorov–Smirnov test, *P* = 9.67 × 10^−23^ for ACM versus joint angle model; *P* = 2.83 × 10^−22^ for ACM versus temporal model; and *P* = 3.91 × 10^−47^ for ACM versus naive skeleton model) as well as the smallest number of error values above 4 cm (ACM, 9.36%; joint angle model, 11.61%; temporal model, 13.72%; and naive skeleton model, 19.12%). Paw placement errors increased the longer a surface marker remained undetected for the ACM and the naive skeleton model (Fig. 2j; linear regression; slope of 1.49 cm s^−1^ and intercept of 1.13 cm for ACM; and slope of 2.77cm s^−1^ and intercept of 1.39 cm for the naive skeleton model) and errors were significantly lower when comparing both models (*P* value using one-sided Mann–Whitney rank test of 3.91 × 10^−47^ for ACM versus naive skeleton model).

### Kinematics of cyclic gait behavior in mice and rats

**Fig. 3:**
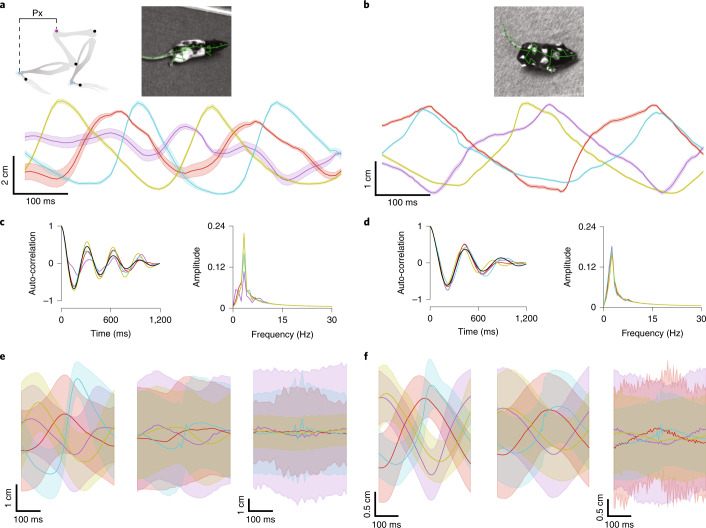
Periodic gait cycles in freely moving rats and mice. **a**, Trajectories in the freely moving rat of the normalized *x* position (Px) as a function of time (mean ± s.d. of 1,000 propagations through the probabilistic model) for the left wrist (purple), right wrist (red), left ankle (cyan) and right ankle (yellow) joint during gait. Schematic above at left illustrates the normalized position of an ankle joint (cyan spot) and normalization joint (magenta). Individual traces show the estimated position (solid line) with the uncertainty in the position represented by the width of the surrounding shaded area. Note that the left and right wrist joints were occluded for large parts of this segment, illustrated by the larger uncertainty in position on these traces. **b**, As in **a**, but for data from a freely moving mouse. All joints are clearly visible throughout the mouse segment, resulting in small uncertainty ranges for all traces. **c**, Autocorrelations of the normalized *x* position for data from a freely moving rat as a function of time (left) for four different limbs as well as a corresponding model fit via a damped sinusoid (black). Fourier-transformed autocorrelations of all limbs (right) have their maximum peak at the same frequency. Colors as in **a**. **d**, As in **c**, but for data from a freely moving mouse. **e**, Population-averaged trajectories of the normalized *x* position for data from freely moving rats as a function of time for the ACM (left), the naive skeleton model (center) and the surface model (right). Individual traces represent mean and s.d. Data from 28 sequences, 146.5 s, 58,600 frames in total from four cameras, *n* = 2 rats. Colors as in **a**. Trajectories of the ACM and the naive skeleton model correspond to the 3D joint locations, whereas trajectories of the surface model correspond to the 3D locations of the associated surface markers. Scale bar on left applies to both left and center. **f**, As in **e**, but for data from freely moving mice. Data from 29 sequences, 93.8 s, 73,536 frames total from four cameras, *n* = 2 mice. Scale bar on left applies to both left and center.

Smooth and periodic reconstruction of an animal’s average gait cycle during walking or running is only possible with robust and accurate tracking of animal limb positions. To establish whether the ACM could generate an average gait cycle from freely moving data, we next extracted individual gait cycles for both the rat (Fig. 3a) and mouse (Fig. 3b) from multiple behavioral sequences (Supplementary Fig. 3 and Supplementary Videos 10–12) where joint velocities exceeded 25 cm s^−1^ (*n* = 2 rats, 28 sequences, 146.5 s and 58,600 frames in total from four cameras; and *n* = 2 mice, 29 sequences, 93.8 s and 73,536 frames in total from four cameras). The ACM-extracted gait cycles for both species were stereotypical and rhythmic (Fig. 3a,b), showing periodicity in autocorrelations of extracted limb movement (Fig. 3c,d; damped sinusoid fit, frequency of 3.14 Hz; decay rate of 2.49 Hz; and *R*^2^ = 0.90 for rat; and damped sinusoid fit, frequency of 2.24 Hz; decay rate of 2.24 Hz; and *R*^2^ = 0.88 for mouse) and also for both species, a common peak for all limbs in Fourier-transformed data (Fig. 3c,d; maximum peak at 3.33 Hz; and sampling rate of 0.83 Hz for rat; and maximum peak at 2.50 Hz; and sampling rate of 0.83 Hz for mouse). For both the rat and mouse, averaged ACM-extracted gait cycles (Fig. 3e,f and Supplementary Figs. 4–7) were significantly less variable than those obtained from the naive skeleton model (Fig. 3e,f) throughout the entire gait cycle (*P* value using one-sided Mann–Whitney rank test of 1.40 × 10^−49^ for rat and 2.03 × 10^−96^ for mouse). When gait cycles were obtained from only tracking surface markers alone via DLC without any form of underlying skeleton (surface model), high noise levels even made the periodic nature of the gait cycles vanish in its entirety for both species (Fig. 3e,f).

### Comparison of inferred with measured kinematics

**Fig. 4:**
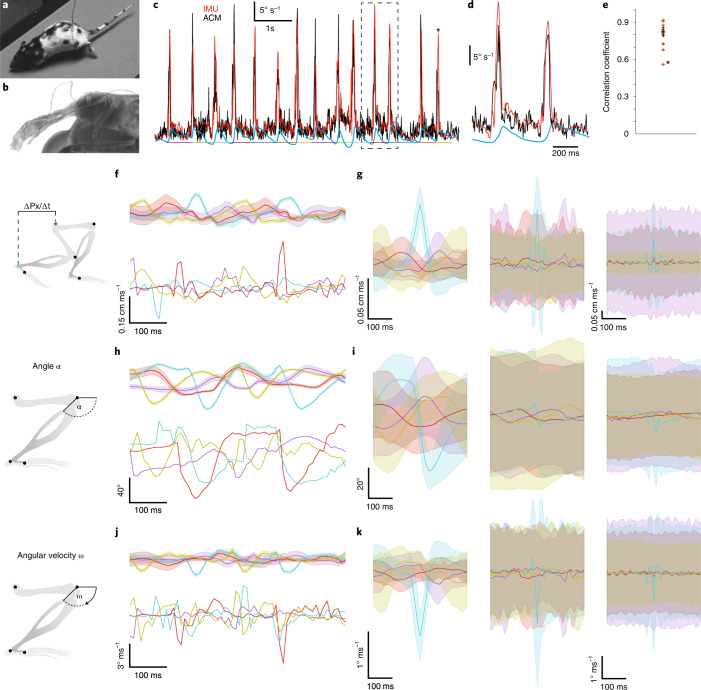
3D pose reconstruction of gait cycles with independent gyroscope-based verification. **a**, Overhead camera image of freely moving rat with attached IMUs and signal wires. **b**, MicroCT image of IMU unit placed on skin over tibia. **c**, Example traces of inferred absolute angular velocity of the leg from the ACM (black), the angular velocity directly measured by the IMU (red) and left ankle *x* position (blue, anatomical position as in **f**). Colored line segments below the traces illustrate the segments used for correlation calculations in **e**. Asterisk marks the peak that corresponds to the lowest correlation value in **e**. Dashed box shown expanded in **d**. **e**, Correlation coefficients between simultaneously recorded ACM (black in **c**) and IMU (red in **c**) traces around peaks (colored segments in **c**) (14 peaks, rat 1, red) and for data from a second rat (blue, 6 peaks). Asterisk indicates the value from the correspondingly marked peak in **c**, vertical black line denotes s.d., horizontal black line denotes the median. **f**, Normalized *x* velocity as a function of time (mean ± s.d. of 1,000 propagations through the probabilistic model) of the left wrist (purple), right wrist (red), left ankle (cyan) and right ankle (yellow) for the ACM (top) and the naive skeleton model (bottom) during gait. **g**, Population-averaged trajectories of the quantities in **f** as a function of time for the ACM (left), the naive skeleton model (center) and the surface model (right). Individual traces represent mean and s.d. Data from 28 sequences, 146.5 s, 58,600 frames in total from four cameras, *n* = 2 rats. Colors as in **f**. Trajectories of the ACM and the naive skeleton model correspond to the 3D joint locations, whereas trajectories of the surface model correspond to the 3D locations of the associated surface markers. Scale bar on left applies to both left and center. **h**,**i**, As in **f** and **g**, respectively, but for the normalized joint angle. Scale bar on left in **i** applies to all three panels. **j**,**k**, As in **f** and **g**, respectively, but for the first temporal derivative of the normalized joint angle (angular velocity). Scale bar on left in **k** applies to both left and center.

We next directly compared limb kinematics inferred by the ACM with the kinematics measured simultaneously from an IMU carried on the same limb below the knee joint (Fig. 4a,b). During gait cycles, the measured absolute limb kinematics matched the ACM-inferred limb kinematics continuously through multiple gait cycles (Fig. 4c,d). Over multiple animals the correlation between the two measurements was high (Fig. 4e; correlation coefficient median of 0.81, s.d. 0.09, *n* = 2 animals) with peak velocities occurring simultaneously (median difference 0.00 s, mean 0.01 s, s.d. 0.11 s, *P* = 0.61, Student’s *t*-test for difference to distribution with mean 0). The reliability of kinematic estimation from the ACM was particularly apparent when comparing joint velocities (Fig. 4f,g), joint angles (Fig. 4h,i), and joint angular velocities (Fig. 4j,k) to the kinematics estimated without the ACM constraints, which were dominated by noise in individual examples (Fig. 4f,h,j), and the cyclic nature of gait was less prominent when compared to traces obtained from the ACM (Fig. 4f,h,j). Consistent with this, ACM-averaged traces (Fig. 4g,i,k and Supplementary Figs. 4–7) had significantly less variance compared to those obtained from the naive skeleton model (Fig. 4g,i,k and Supplementary Figs. 4–7) for all metrics (rat and mouse *P* values using one-sided Mann–Whitney rank test of 2.28 × 10^−55^ and 9.63 × 10^−107^ for velocity; 1.42 × 10^−55^ and 1.63 × 10^−94^ for angle; and 1.44 × 10^−55^ and 7.73 × 10^−108^ for angular velocity, respectively). Cyclic, gait-related, peaks were barely discernible when tracking surface markers only without any form of underlying model skeleton (Fig. 4g,i,k and Supplementary Figs. 4–7), with little if any consistency apparent between individual traces. Additionally, the periodicity of the gait cycles in the form of equidistant peaks was more variable for all metrics for the naive skeleton model than for the ACM (Table 1). Together this shows that the ACM can objectively extract behaviors such as gait, from freely moving animals and quantify complex relationships between limb bones by inferring 3D joint positions over time as well as their first derivatives.

**Table 1:** Temporal periodicity of gait cycles

	ACM		Naive skeleton model	
	Mean (ms)	s.d. (ms)	Mean (ms)	s.d. (ms)
**Rat**				
Position (min. peaks)	75.00	29.01	64.16	56.78
Velocity (max. peaks)	78.33	10.67	80.83	54.99
Angle (max. peaks)	78.33	23.74	74.16	33.53
Angular velocity (min. peaks)	75.00	10.40	53.33	47.78
Total peaks	12		12	
Sampling rate (Hz)	100		100	
**Mouse**				
Position (min. peaks)	82.48	59.57	82.48	67.20
Velocity (max. peaks)	99.91	14.94	97.79	35.58
Angle (max. peaks)	100.77	16.02	85.46	68.39
Angular velocity (min. peaks)	105.44	22.08	97.79	18.02
Total peaks	12		12	
Sampling rate (Hz)	196		196	

### Kinematics of complex behavior

**Fig. 5:**
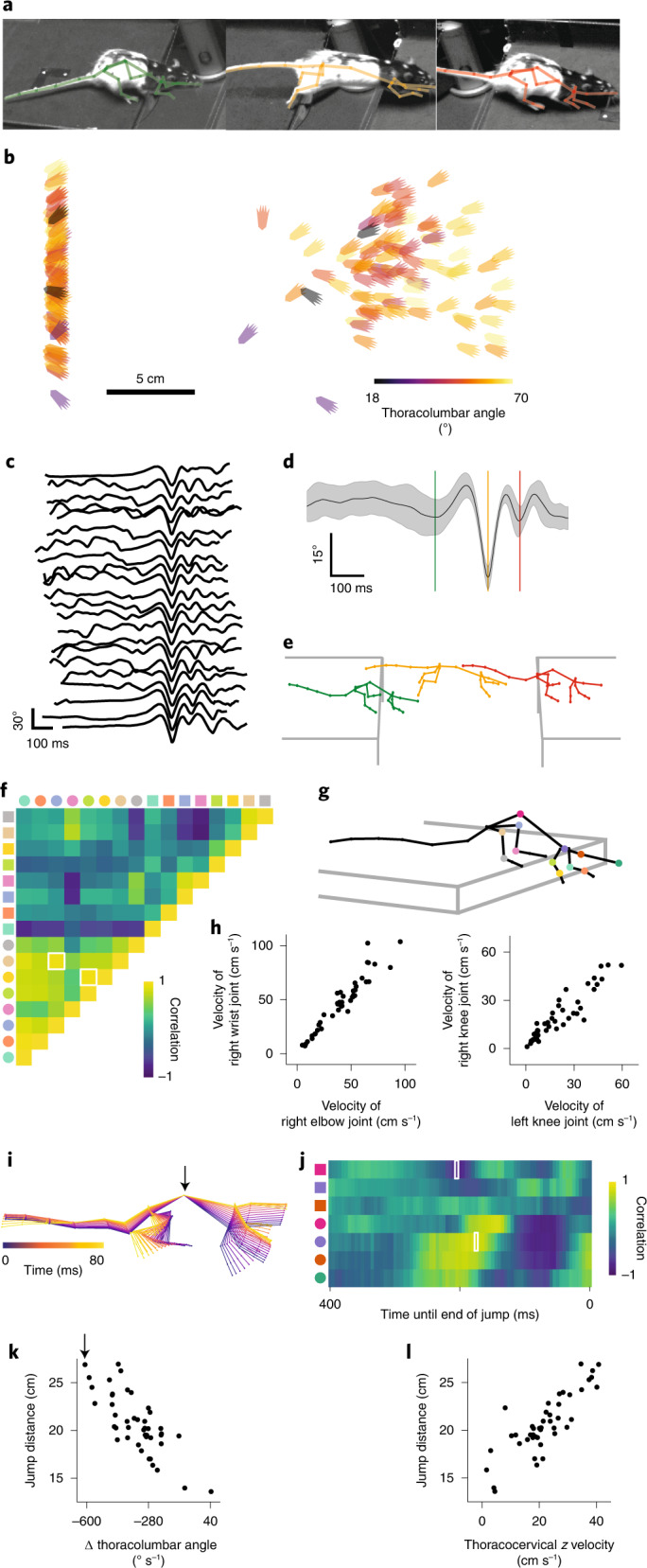
3D pose reconstruction of skeletons allows for detailed quantification of complex behavior. **a**, Images of a rat performing a trial in the gap-crossing task. **b**, Reconstructed *xy* positions of the hind paws at the start and end of the jump color-coded by the joint angle of the thoracolumbar joint for each gap-crossing event of the population. **c**, Averaged joint-angle traces (spine and hind limb joint angles) from 22 out of 44 jump trials. **d**, Joint-angle trace averaged across joints and all jump trials (mean ± s.d.). **e**, Average poses at the start (green), midpoint (orange) and end point (red) of the jump from all jump trials. The three different time points are indicated by colored lines in **d**. **f**, Cross-correlation of the spatial and angular velocities of the limb joints at the start point of a jump. Different marker shapes indicate whether rows or columns represent spatial or angular velocities (circles and squares, respectively). Marker color corresponds to joint markers in **g**. **g**, Average pose at the start of a jump calculated from all jump trials. Joint colors are consistent with the marker colors in **f** and **j**. **h**, High correlation examples for spatial velocities of different limb joints as a function of each other for both animals. The data shown represent the correlation values highlighted in white in **f**. **i**, Overlaid poses of a single animal 240 ms to 160 ms before the end of a jump. Arrow indicates the thoracolumbar joint. **j**, Correlations of the *z* and angular velocities of the head and spine joints for time points up to 400 ms before the end point of a jump. Marker conventions as in **f**. **k**, Jump distance as a function of angular velocity of the thoracolumbar joint for both animals 205 ms before the end of the jump. Poses corresponding to the single data point highlighted with the arrow are shown in **i**. Displayed data represents the correlation value highlighted with a white rectangle in **j**. **l**, Jump distance as a function of *z* velocity of the thoracocervical joint for both animals 175 ms before the end of the jump. Displayed data represent the correlation value highlighted with a white rectangle in **j**.

We next used the ACM to analyze motion kinematics and segment a more complex decision-making behavior, the gap-crossing task, in which distances between two separate platforms were changed forcing the animal to re-estimate the required jumping distance (Fig. 5a). Reconstructed poses during gap-estimation and jump behaviors consisted of sequences where animals either approached or waited at the edge of the track before jumping (*n* = 42; Supplementary Fig. 8 and Supplementary Videos 13 and 14) or reached with a front paw to the other side of the track before jumping (*n* = 2; Supplementary Videos 15 and 16). Hind paw positions could be inferred throughout the jump and compared to skeletal parameters during the behavior (Fig. 5b; 44 trials, *n* = 2 animals). As rats jumped stereotypically, we used the ACM to objectively define decision points in the behavior, such as time of jump, from each individual trial. Averaging spine segment and hind limbs joint angles around the time of the jump, giving a single joint-angle trace, provided a metric with a global minimum (Fig. 5c) during the jump that was independent of how the animal crossed the gap (Supplementary Fig. 9). This enabled objective identification of jump start, midpoint and end point from each individual jump. We averaged traces of joint angles across joints and trials to generate average ACM poses (Fig. 5d,e). Autocorrelations for spatial and angular limb velocities allowed quantification of the interdependency of joint movements at any point within the jumping behavior, for example at the start point of a jump (Fig. 5f,g). This displayed a significant correlation between the spatial velocity of the right elbow and wrist joints (Fig. 5h; Spearman correlation coefficient of 0.95; two-tailed *P* value testing non-correlation of 5.40 × 10^−24^), as well as joint interactions across the midline, such as a significant correlation between spatial velocity of the right and left knee joints (Fig. 5h; Spearman correlation coefficient of 0.93; two-tailed *P* value testing non-correlation of 6.79 × 10^−20^). As the animals jumped across the gap, changes in the bone angles and their derivatives (Fig. 5i) were correlated with distances that the animals jumped (Fig. 5j). For example, angular velocities of the thoracolumbar joint and vertical velocities (*z* velocity) of the thoracocervical joint were significantly correlated with jump distance 205 ms and 175 ms, respectively before the animals landed (Fig. 5k,l; Spearman correlation coefficient of −0.73 and two-tailed *P* value testing non-correlation of 1.13 × 10^−8^; and Spearman correlation coefficient of 0.81 and two-tailed *P* value testing non-correlation of 1.12 × 10^−11^).

## Discussion

We developed an ACM for tracking skeletal kinematics of untethered freely moving rats and mice, at the resolution of single joints, which enabled the quantification of joint kinematics during gait and gap-crossing behaviors. From these kinematic measurements, the ACM was able to build a comprehensive comparative map of the kinematic sequences throughout decision-making behaviors that could be compared to the behavioral outcome. Accurate generation of skeletal kinematics relied on incorporating skeleton anatomy, requiring smoothness of rotations and imposing motion restrictions of joints^9^, as animal poses are limited by both bone lengths and joint angle limits^9^. The joint angle limits used in the ACM were taken from data measured from cats; however, comparative studies measuring quadruped gait cycle have shown a remarkable similarity for the limb, pelvis and scapula angles during the different phases of the gait cycle^13^. Given that the joint angle limits used for the ACM only prevented un-natural angles from occurring, we used the most detailed dataset available. We generated ground-truth data to quantify both the accuracy of the algorithm used to fit the model skeleton to the behavioral data and also the performance of the ACM at estimating limb and joint trajectories. In addition, inferred limb kinematic accuracy was verified by direct measurement of limb angular velocity using limb-mounted IMUs. While the IMUs alone do not directly measure bone position, but instead kinematics, taken together with the MRI and frustrated internal reflection-generated ground-truth comparisons to algorithm inferred kinematics, we show that the ACM accurately quantifies limb kinematics during cyclic gait behaviors and more complex behaviors.

Our approach ushers in a suite of possibilities for studying the biomechanics of motion during complex behaviors in freely moving animals and complements developments in detailed surface tracking^36^ with the expectation that the ACM approach will also work for other small animals such as ferrets and tree shrews. This approach also opens up future investigations to model forces applied by tendons and muscles^9,51^ and starts bridging the gap between neural computations recorded in freely moving animals^52–55^ and the mechanistic implementation of complex behavior^56–58^. This approach complements advances in pose estimation by the ability to accurately infer joint kinematics in freely behaving animals. Traditionally, single-plane X-ray-based cineradiography and fluoroscopy approaches have been used to calculate joint kinematics^11–14,16^ and recently in 3D^17–19,23,24^ across multiple animal species^20–22^. While these approaches directly measure bone positions as the animal behaves, the spatial area that can be observed and the exposure time to radiation is limited thereby also limiting the number of joints that can be simultaneously imaged as well as the range of observable behaviors^23,24^. On the other hand, inferring bone rotations and positions using surface imaging alone is complicated in animals covered in fur as the spatial relationship between skeleton and overlying soft tissues are less apparent^12,30–32^. Surface markers can be rationally placed around joints, which in rodents would otherwise be problematic to locate reliably. By linking multiple surface markers to individual joints, the ACM approach reduced potential errors in joint position estimates due to movement of the skin relative to the joint.

Deep neural networks have been used to approach the problem of detecting an animal’s pose in the form of 2D features from an image without anatomically constrained skeleton models^27,33,34^. The 3D poses can be inferred from these 2D features by means of classical calibrated camera setups^59^; however the 2D detection in one camera image does not benefit from the information from other cameras and the triangulation may suffer from resulting mislabeling of 2D features as well as missing detections due to occluded features. A recent approach^38,60^ overcomes many of these issues by mapping from recorded images directly to 3D feature locations, again using deep learning, and is capable of classifying animal behaviors across many species^38^. An alternative approach is to use measurements that directly yield 3D information, for example RGBD^61^. In parallel, there has been substantial developments in pose estimation of humans, including the possibility to track multiple individuals in real time^60,62–64^, some of which include explicit models of kinematics^65,66^. In general, these approaches triangulate joint positions of readily detectable key points in the images, which has the advantage of not requiring application of surface markers. Geometrical constraints and prior knowledge dynamics can be included, for example through using pictorial structures or deformable mixtures of parts^28,67^. Triangulation of joints works well when the relation between the joint and its surface representation are well defined and recognizable, as is the case for humans and insects^28^, but is not necessarily so successful for animals such as mice and rats, where surface representations for many joints are not as clearly defined or visible^12,30–32^. The ACM uses a probabilistic framework to infer latent variables for joint position from 2D markers that are in different spatial positions than the joints, which also allowed the incorporation of prior knowledge and constraints on joint angles. A similar approach was taken by GIMBAL^68^, which uses a hierarchical probabilistic model of a rigid skeleton and infers parameters with Bayesian sampling approaches. In contrast to studies that target pose estimation, skeletons and kinematics inferred by the ACM were validated with ground-truth measurements obtained using MRI and IMUs. The ACM uses DLC^34^, an existing method, to detect 2D anatomical markers and inferred 3D positions and kinematics of movement with an RTS smoother based on anatomical constraints and mechanistic knowledge of bone rotations^9,51^, considering the trajectory of 3D positions over time. One disadvantage of the ACM is that the RTS smoother is computationally expensive, which currently prohibits real-time inferring of skeletal kinematics in freely behaving animals^27,69^. A second disadvantage is that it currently uses a simplified skeleton model; for example, it does not model all joints of the vertebra and also does not include a detailed model of the digits.

We expect future work in the field of animal pose estimation to combine both supervised learning techniques^36,38^ and mechanistic model constraints^9,51^, to simultaneously capitalize on their different strengths, for example by applying a smoother with anatomical knowledge such as the ACM directly to 3D positions from an image-to-3D framework^38^. Our approach has the capacity to extend existing methods and not only to enhance the detail in which animal behavior can be studied and quantified, but it also provides an objective and accurate quantification of limb and joint positions for comparison with neuronal recordings.

## Methods

### Obtaining video data of behaving animals

All experiments were performed in accordance with German guidelines for animal experiments and approved by the Landesamt für Natur, Umwelt und Verbraucherschutz, North Rhine-Westphalia, Germany. Nine Lister hooded rats (Charles River Laboratories), weighing 174 g (rat no. 1), 178 g (rat no. 2), 71 g (rat no. 3), 72 g (rat no. 4), 735 g (rat no. 5), 699 g (rat no. 6), 189 g (rat no. 7), 228 g (rat no. 8) and 214 g (rat no. 9) and four mice weighing 36 g (mouse no. 1), 35 g (mouse no. 2), 33 g (mouse no. 3) and 27 g (mouse no. 4) were used. Anatomical landmarks for tracking limb and body positions consisted of black or white ink spots (5–8 mm diameter; black markers, Edding 3300; and white markers, Edding 751; Edding) that were painted onto the fur in a stereotypical pattern near-symmetrical around the animals’ mid-sagittal axis (Supplementary Fig. 1b). Anatomical markers were applied under anesthesia with isoflurane (2–3%) with body temperature maintained at around 37.5 °C using a heating pad and temperature probe. In some experiments with rats (animal nos. 7–9), custom-assembled IMUs (see ‘Assembly of inertial measurement units’ below) were fixed to the middle of the dorsum on the foot and the upper leg at approximately the level of the center of the femur on either the left or right side using biologically inert silicone (KwikSil, WPI), with the associated fine cabling led up the leg and fixed to the fur just lateral of the spine with the same silicone so as to not interfere with the animals leg movement. Subsequently, animals were allowed to recover for approximately 45 min before datasets were acquired in an open arena and/or gap-crossing track. The open arena was 80 × 105 cm^2^ with 50-cm-high gray walls. The gap-crossing track consisted of two 50 × 20 cm^2^ platforms with 2.5-cm-tall walls (except jump-off edges), mounted 120 cm off the ground on a slide mechanism along the long edge to allow manual adjustment of the distance between the platforms from 0 to 60 cm. The floor was covered with neoprene material for secure grip for the animals’ feet. A water delivery spout was located at one end of the track. To encourage gap-crossing behavior, animals were water-restricted (full access to water 2 d per week, otherwise only on the gap-crossing track). After each successful crossing of the gap, 50–100 µl water was available at the spout. Animals received a minimum of 50% of their daily ad libitum water consumption either during the training or recording sessions or as a supplement after the last session of the day. Gap-crossing training of two daily sessions commenced approximately 2 weeks before the recording. Gap distances were pseudo-random, but reduced in cases where the animal refused to cross. Recordings with simultaneous IMU acquisition were performed on a raised open track with dimensions 105 × 28 cm (length × width) with 3-cm-high walls on the long edges and 24-cm-high walls at the short ends. The open arena for mice had dimensions of 45 × 50 cm (length × width) without surrounding walls, elevated off the floor by 145 cm. Both setups were homogeneously illuminated using eight 125-cm-long white LED strips with 700 lm m^−1^ (PowerLED), arranged equidistantly in a patch of 125 × 80 cm^2^ and 125 × 55 cm^2^ at a distance of 130 cm and 150 cm above the ground of the open arena and the gap-crossing track. For mice, two additional 100-cm-long LED strips were used on opposing sides of the arena at a distance of approximately 35 cm to minimize shadowing of the feet. Datasets were acquired using four synchronously triggered cameras (ace acA1300-200um, Basler, 1,280 × 1,024 px^2^) above the setups, covering all parts of the setup by at least two cameras, with the majority covered by all four. Videos were recorded at 100 Hz (gait dataset) and 200 Hz (gap-crossing, FTIR). Animals’ foot positions were quantified using a custom-made FTIR plate of 60 × 60 cm^2^, with an IR-LED strip (Solarox 850 nm LED strip infrared 850 nm, Winger Electronics) mounted along the edges such that IR light could propagate through the plate from two opposing sites. Paw placements were recorded using two additional cameras (ace acA1300-200um, Basler, 1,280 × 1,024 px^2^), synchronized with the overhead cameras, mounted underneath the plate and equipped with infrared-highpass filters (Near-IR Bandpass Filter, part BP850, useful range 820–910 nm, FWHM 160 nm, Midwest Optical Systems).

### Obtaining MRI scans to evaluate learned skeleton models

To locate labeled surface markers, custom-made MRI markers (premium sanitary silicone DSSA, Fischerwerke) were attached to the respective positions on the surface of the animals’ bodies. Post mortem MRI imaging in six rats and four mice was performed at a field strength of 3 T (Magnetom Prisma, Siemens Healthineers), using the integrated 32-channel spine coil of the manufacturer and a 64-channel head coil, respectively. The data were acquired using a 3D turbo-spin echo sequence with variable flip-angle echo trains (3D TSE-VFL).

Detailed rat MRI protocol parameters for 3D TSE-VFL imaging with a turbo factor of 98 were as follows: 3,200 ms repetition time, 284 ms effective echo time, 586 ms echo train duration and 6.3 ms echo spacing using 300 Hz per px readout bandwidth for one slab with 208 slices covering the whole rat at 0.4 × 0.4 × 0.4 mm^3^ isotropic resolution. One average in combination with parallel imaging (here GRAPPA acceleration factor of 2) yielded an overall acquisition time of 18 min 5 s.

Mouse MRI images were collected utilizing the following parameters: 0.3 × 0.3 × 0.3 mm^3^ isotropic resolution, 605 ms echo train duration, 6.72 ms echo spacing, 309 Hz per px readout bandwidth and 15 min 34 s total scan time, ceteris paribus.

### Assembly of inertial measurement units

IMUs (MPU-9250, TDK InvenSense) for independent measurement of limb motion were connected without a circuit board using twisted pairs of 50-µm enameled copper wires, with decoupling capacitors on the supply lines for each IMU. The IMUs were embedded in electronic-component-embedding silicone (Magic Rubber, Raytech) for protection and connected to an external microprocessor board (Teensy 3.6, PJRC) that received a frame synchronization signal from the overhead cameras, which triggered a 1-ms pulse to the IMU’s FSYNC input and streamed the data to a computer via USB.

### Calibrating multi-camera setups

We based the calibration of multiple cameras on a pinhole camera model with second-order radial distortions and OpenCV^70^ functions for the detection of Charuco boards. Calibration was performed via OpenCV calibration functions and subsequently optimized for reprojection error (Supplementary Text).

### Defining a 3D skeleton model

The generalized skeleton model was modeled as a graph with joints as vertices and one or multiple bones as edges. (Supplementary Fig. 1a). Front limbs were modeled as four edges, representing clavicle, humerus, radius and ulna, and metacarpal and phalanges. Associated vertices corresponded to the shoulder, elbow and wrist, with the last vertex representing the tip of the middle phalanx. Hind limbs were modeled as five edges representing the pelvis, femur, tibia and fibula, tarsus and phalanges, with the associated vertices representing the hip, knee, ankle and metatarsophalangeal joints, with the last vertex representing the tip of the middle tarsal. The tail was modeled as five edges and five vertices, with the last vertex representing the tip of the tail. The spine was modeled as four edges, representing the cervical, thoracic and lumbar spinal regions and the sacrum, with three intervening vertices. The head was modeled as a single edge, with a vertex at the tip of the nose, and a second vertex representing the joint to the first cervical vertebra. In resting pose, all bone rotations were set to zero, such that all edges (bones) pointed toward the positive *z* direction of the world coordinate system, except the clavicle to collarbone and sacrum to pelvis edges which pointed perpendicularly in the *x*–*y* plane (Supplementary Fig. 1a), which was also kept constant during pose reconstruction. Each edge was further equipped with a local coordinate system originating at the start joint (for example the left shoulder joint vertex for the left humerus edge), with the edge pointing along the *z* direction and *x* and *y* directions such that rotations around the *x* direction were equivalent to flexion and extension, rotations around the *y* direction to abduction and adduction and a rotation around the *z* direction with internal and external rotation.

### Constraining poses based on joint angle limits

We implemented joint angle limits based on measured minimum and maximum values for flexion or extension, abduction or adduction and internal or external rotation in domestic house cats^43^, due to the unavailability of rat or mouse data. For vertices approximating head, spine or tail joints, due to a lack of data, we assumed angle limits for rotations around the *x* and *y* direction as ±90° and no capacity to rotate around the *z* direction. Thus, the respective child vertex may be placed in any point of a hemisphere with a radius of the length of this edge. Angle limits were enforced for each axis individually, without considering co-dependence (applied to the individual entries of the Rodrigues vector). Joint angle limits were then established after adjustment from the literature pose to our resting pose as shown in Supplementary Table 1.

### Constraining surface-marker positions based on body symmetry

When learning surface-maker positions and bone lengths we reduced the number of free parameters by assuming symmetry in both marker placement and physiology in the *y*–*z* plane. Thus, for any bilateral marker, we only optimized one side with box constraints to the respective side and inferred the other side by mirroring on the *y*–*z* plane. Supplementary Text provides a table of box constraints (Supplementary Fig. 1). The upper bound of the left-sided surface marker on the shoulder in *z* direction for the two large rats (animal nos. 5 and 6), which was also set to 0 to prevent the bone lengths of the collarbones becoming zero during learning.

### Constraining bone lengths based on allometry

We applied loose constraints on the length of limb bones based on published data. For rat skeletons, we used a linear relationship between body weight and bone lengths^44^.

For mice, equivalent proportionality factors were not available, but as all mice used were adult and had attained a fully adult size and weight, we based the limb length constraints for the bones on published bone lengths measured from microCT data^42^. In both cases we chose ±10 × s.d. as box constraints. Bone length constraints used are shown in Supplementary Table 2. For bones that were not part of the limbs, no constraints were enforced. As for the markers, we assumed symmetry and only optimized a common single length for bilateral bones.

### Learning bone lengths and surface-marker positions

To learn bone lengths and surface-marker positions we simultaneously fitted our generalized 3D skeleton model to manually labeled 2D positions of surface markers at different time points for each animal. For this, we utilized the L-BFGS-B algorithm^71^, minimizing the 2D distance between manual labels and projected 3D positions into each camera. We simultaneously optimized all per-frame pose parameters (position and bone rotation) and the skeletal parameters (bone length and relative position of markers to joints), which remain constant over time and define the final individual skeleton, for the subsequent pose estimation of the animal (Supplementary Video 1). In initial experiments, we used 300-s long sequences of freely behaving animals recorded via four different cameras with a frame rate of 100 Hz and labeled every 50th frame in each camera totaling 2,400 training frames at 600 different time points. For rat IMU and mouse datasets we used all available manually labeled data from the DLC training (stated below in ‘Training deep neural networks to detect 2D locations of surface markers’). Bone lengths were initialized by the mean of their upper and lower bounds or zero when there were no constraints and surface-marker positions were initialized to be identical to the joints they were attached to. Poses were initialized to the resting pose but global skeleton locations and rotations were adjusted before the fitting to loosely align with the locations of an animal’s body, as seen by the cameras. Once values for bone lengths and surface-marker positions were learned, we used them for all further pose reconstructions of the respective animal.

### Comparison of skeleton parameters with MRI data

To estimate the quality of the skeleton estimation, we compared the distances between adjacent joints (‘bone lengths’) between the learned positions from ACM and manually marked joint positions in MRI scans for each animal (Fig. 1f). To determine the 3D positions of the respective spine joints in the MRI scan, we counted vertebrae such that each modeled spine segment matched its anatomical counterpart with respect to the number of contained vertebrae^40^. All ground-truth joint positions, except those for the metatarsophalangeal joints, could be identified manually in the MRI scan (four joint locations in total). These missing locations were assumed to be identical to the positions of the corresponding metatarsophalangeal markers. The joints used for the comparison are shown in Supplementary Table 3.

### Performing probabilistic pose reconstruction

For probabilistic 3D pose reconstruction we implemented an unscented RTS smoother^45,46^, whose fundamental principles are based on the ordinary Kalman filter formulation^47^, but can consider both past and future and be used to perform probabilistic pose estimation in a nonlinear state space model, as our formalism, for example, introduces nonlinearities through the usage of trigonometric functions in bone rotations. In this approach, described in mathematical detail in the Supplementary Text, time series data are modeled as a stochastic process generated by a state space model, where at each time point hidden states give rise to observable measurements and fulfill the Markov property (each hidden state only depends on the preceding one; Supplementary Fig. 10). This formalism allowed us to represent each pose as a low-dimensional state variable, corresponding to the location and the individual bone rotations of a reconstructed skeleton (dimension of hidden state variable was 50; 3 variables for 3D location of the skeleton plus 47 variables for bone rotations). The measurable 2D locations of surface markers (which were given by the outputs of the trained neural network) had a higher dimensionality and were represented via measurement variables (dimension of measurement variable, maximal 344; 43 surface markers times four cameras times two variables for the 2D location of a surface marker). We assumed the hidden states to be (conditionally) normally distributed, where temporal constraints are implicitly modeled through the transition kernel of the Markov process (the probabilistic mapping between one state and the next). We learned the unknown model parameters (the initial mean and covariance of the state variables as well as the covariances of the transition and measurement noise) via an EM algorithm^48^ (maximal 2,944 model parameters in total; 50 parameters for mean of initial hidden state variable plus 1,275 parameters for covariance matrix of initial hidden state variable plus 1,275 parameters for covariance matrix of transition noise plus maximal 344 parameters for diagonal covariance matrix of measurement noise), which aims to maximize a lower bound of the state space model’s evidence, the evidence lower bound (ELBO), accounting for each pose within a behavioral sequence. This is achieved by alternating between an expectation step, in which we obtain the expected values of the state variables given a fixed set of model parameters via the unscented RTS smoother, and a maximization step, in which these model parameters are updated in closed form to maximize the ELBO^72^. After convergence of the EM algorithm, final poses were obtained by applying the unscented RTS smoother using the learned model parameters.

### Accounting for missing measurements during pose reconstruction

To account for missing 2D positions of surface markers, for example due to marker occlusions or lack of detection confidence, we modified the plain unscented RTS smoother formulation and the EM algorithm by accordingly zeroing rows and/or columns of measurement covariance matrices during the filtering path of the smoother^73,74^ and when maximizing the ELBO (Supplementary Text).

### Enforcing joint angle limits during pose reconstruction

The plain formulation of the unscented RTS smoother does not account for box constraints for bounding state variables representing bone rotations. To still allow for anatomically constrained pose estimation we instead optimized unbound state variables, which were mapped onto the correct lower and upper bounds for joint angle limits via sigmoidal functions (error functions) (Supplementary Text). These functions had slope one at the origin and were asymptotically converging toward the lower and upper bounds of the respective joint angle limits.

### Evaluating the influence of anatomical and temporal constraints

To evaluate the influence of constraints, we determined poses using skeleton models employing both anatomical and temporal constraints (ACM), only one type of constraint (anatomical or temporal) or no constraints (naive skeleton model). In models without anatomical constraints, all constraints except tight (0°,0°) were relaxed to (−180°,180°), effectively allowing the full solid angle range. Pose parameters for these two models were initialized by fitting the pose of the first time point of a behavioral sequence to auto-detected 2D markers equivalently to how the skeleton parameters were learned. The covariance matrices for the initial state variables and the state and measurement noise learned via the EM algorithm were initialized as diagonal matrices with 0.001 in all diagonal entries and the off-diagonal values kept constant for the measurement noise covariance matrix during the maximization step of the EM algorithm.

In models without temporal constraints, the unscented RTS smoother was discarded and instead, each pose was fitted individually as above, initialized by the previous frame.

### Evaluating pose reconstruction accuracy via a FTIR touch-sensing system

Ground truth for the FTIR analysis was obtained by manual labeling of every 40th frame. In the overhead camera images, paw centers and three individual fingers and toes were manually labeled for each limb, whereas in the underneath camera images only finger and toe silhouettes were labeled and paw centers were identified as the interpolated intersection of the three fingers or toes. Silhouette *x*–*y* coordinates were then determined by the intersection of the camera ray corresponding to the respective image coordinate and the surface of the transparent floor. Velocity and acceleration values for the four different models were derived from central eighth-order finite differences based on the reconstructed 3D positions of the metatarsophalangeal or wrist and finger or toe markers. Paw position errors of undetected markers were obtained by only using paw position errors of surface markers that were not detected by the trained neural network (confidence <0.9).

In the analysis of accuracy degradation after the last successful detection, the backward and forward connection of the unscented RTS smoother was addressed by selecting the respective minimal temporal distance to the previous and next successful detection, whereas for the joint angle and naive skeleton models only the previous was considered. For the resulting analysis we only included errors for which the corresponding sample size was at least ten.

### Analyzing gait data

To extract gait periodicity, we considered 3D joint locations relative to the joint that connects lumbar vertebrae with the sacrum and with the *x* direction set as the anteroposterior axis defined by the new origin and the joint linking cervical with thoracic vertebrae. Angles, positions and translational and rotational velocities were calculated in this coordinate system, where bone angles were defined as the angle between the new *x* direction and a respective bone. To model autocorrelations of *x* positions, we fitted damped sinusoids the four different traces of each limb via gradient decent optimization. For population averages of *x* positions, bone angles and their temporal derivatives, we detected midpoints of swing phases by identifying maximum peaks of *x* velocities above 25 cm s^−1^. Individual traces were extracted containing data up to ±200 ms around each peak, aligned by the peak and averaged across the entire population. In case of the pure surface-marker tracking, 3D positions where triangulated based on the two DLC 2D detections with the highest confidence values.

### Analyzing IMU data

For comparison of directly gyroscope-measured angular velocity and the absolute angular velocity of the tibia derived from ACM, IMU and videography data were synchronized by triggering acquisition times on the IMU with a synchronization signal derived from the overhead imaging cameras. Both the exposure active signal from the overhead cameras and the IMU synchronization signal were recorded with a multi-line analog to digital converter (Power 1401, Cambridge Electronic Design). The angular velocity of the left tibia in frame n was derived by calculating the angle of the rotation matrix that transforms its absolute orientation in space in frame n into the orientation in frame *n* + 1, and dividing by the time period of one frame. Gait periods were segmented via the minima of normalized *x* positions of the left ankle (see ‘Analyzing gait data’ section) identified with a 120-frame minimum filter, and the individual Pearson correlation coefficients of the resulting segments of the IMU and ACM angular velocity trace were calculated. For the analysis of peak differences, maxima in the IMU and ACM traces were identified with a 120-frame maximum filter and each IMU trace maximum was associated with the closest ACM trace maximum.

### Analyzing gap-crossing data

Each of the 44 gap-crossing sequences was 1 s long and contained 200 frames per camera, totaling 35,200 frames. Due to the limited number of gap-crossing events and recorded frames, we used 20% of the frames to train the neural network (we took every fifth frame of the recorded gap-crossing sequences for its training). Velocity values were derived from eighth-order central finite differences of reconstructed 3D joint positions and joint angles were defined as the angle between two connected bones. To obtain start, mid and end points for each jump we averaged joint angles of all spine and hind limb joints. The averaged metric was characteristic for each jump; distinct peaks were always present in the following order: local minimum, local maximum, global minimum, local maximum and local minimum. We defined the start and end point of each jump as the first and last local minimum of this sequential pattern. Resulting jump start and end points were in close agreement with those obtained from manual assessments of gap-crossing sequences by a human expert. Jump distances were calculated as the absolute *x*–*y* difference of the average of the ankle, metatarsophalangeal and toe joint positions, at the start and end point of each jump. To obtain population-averaged poses for the jump start, mid and end points, we aligned all poses at the given time points and calculated characteristic jump poses by averaging them across the entire population. For the population-averaged mean angle traces we aligned each individual trace according to the mid-point of each jump and then averaged these across the entire population. For further analysis, jump distances were correlated with spatial *z* velocities and angular velocities of spine joints at time points up to 400 ms before the end of a jump and absolute spatial velocities and angular velocities of hind limbs joints were correlated with each other at the start point of a jump.

### Statistics

All *P* values were calculated across imaging frames, ignoring correlations across frames.

### Computing hardware

All pose reconstructions and analyzes, including DLC training and detection, were either conducted on a workstation equipped with an AMD Ryzen 7 2700X CPU, 32 GB DDR4 RAM, Samsung 970 EVO 500 GB SSD and a single NVIDIA GeForce RTX 1080 Ti (11 GB) GPU or a cluster node equipped with two Intel Xeon Platinum 8268, 768 GB RAM and four NVIDIA Quadro RTX 6000 (only one used by algorithm) using Ubuntu 18.04.5 LTS, Ubuntu 20.04 LTS and CentOS Linux release 7.9.2009.

### Software

The implementation of the ACM pipeline was written using Python v3.7.6 and the following packages: autograd v1.3, cudnn v7.6.5, numpy v1.18.1, jax v0.2.0, pytorch v1.4.0, scipy v1.4.1, tensorboard v1.14.0 and tensorflow v1.14.0.2.

#### Summary of ACM data-processing steps

1. Manual-labeling initialization step
  Manual labeling of marker points using ACM-traingui (https://github.com/bbo-lab/ACM-traingui).
  This step provided the data for training the DeepLabCut network and verified tracked surface-marker positions for learning the model skeleton (see ‘ACM (pose estimation) step’ below). Marker points were manually labeled by clicking on the 2D position of each surface marker in each camera view. Surface markers were assigned specific labels such as ‘ankle (left)’ to facilitate anatomical relationships for the surface markers. Datasets presented in this manuscript had between 180 and 600 manually labeled time points (with four cameras, 720–2,400 labeled images). Manually labeled images covered a wide variety of different animal poses.
  As a verification step, we used the camera calibration to triangulate the labeled markers in space and thereby calculate the reprojection error. Reprojection errors above 5 px were manually checked and corrected as necessary.
2. DeepLabCut (DLC) step
  Detection of 2D positions of marker points in all camera frames using DLC (via https://github.com/bbo-lab/ACM-dlcdetect; duration of approximately 35 h on single cluster node).
  DLC was first trained on the manually labeled data and subsequently extended the labeling to all frames in the desired segment (segments in the current study were multiple 10,000 s of frames for each camera). DLC detected the 2D position of the markers and corresponding labels in each frame for each camera independently (without taking angles from other cameras and the camera calibration into account).
  DLC also provided a certainty value for marker detections. Here we used labels with a certainty value of 90% and above.
3. ACM (pose estimation) step
  Pose estimation from the DLC-labeled 2D marker positions using the ACM (https://github.com/bbo-lab/ACM; duration of approximately 2 h for a segment of 10 s on single cluster node).
  The ACM output bone lengths and joint angles, from which the full pose with 3D joint positions were derived, ran in two steps:
  For learning the model skeleton, a model skeleton (the bone lengths and bone positions relative to markers) was learned from a subset of frames (further details are in section 3 ‘Skeleton model’ in Supplementary Text). As correct labeling is particularly important, we used the manually labeled and checked data from step 1 for this purpose. Note that frames in which the surface markers were automatically detected could be used here instead.
  For pose estimation, the respective pose for each time point was estimated from 2D marker positions detected by DLC using the EM algorithm (further details are in section 4 ‘Probabilistic pose estimation’ in Supplementary Text).

### Reporting summary

Further information on research design is available in the Nature Research Reporting Summary linked to this article.

## Online content

Any methods, additional references, Nature Research reporting summaries, source data, extended data, supplementary information, acknowledgements, peer review information; details of author contributions and competing interests; and statements of data and code availability are available at 10.1038/s41592-022-01634-9.

## Supplementary information

Supplementary Information Supplementary Figs. 1–12, Supplementary Text and Supplementary Tables 1–3.

Reporting Summary

Supplementary Video 1 Schematic video of how a skeleton pose is adjusted. First the entire skeleton is translated, then individual rotations are applied to each bone to obtain a new pose, which also changes the 3D locations of joints and attached surface markers. Last, the new 3D surface-marker positions are projected onto the four different cameras to obtain 2D locations.

Supplementary Video 2 Example of tracking performance on normal rearing behavior displayed by an exploring rat.

Supplementary Video 3 Video of recorded frames from one underneath camera used in the FTIR imaging setup. Locations of reconstructed paw positions (blue dots) as well as their corresponding uncertainty values (red point clouds; ‘large’ point clouds indicate high uncertainty) are shown for all four limbs. The frame rate is 20 Hz.

Supplementary Video 4 Example video of a behaving small animal taken from the FTIR dataset, with skeletal pose reconstructions from the four different models. Color-coding of the skeletons indicates uncertainty values for the individual joints and bones for the full and temporal model (purple to yellow color indicates low to high uncertainty), with high uncertainty values highlighted (red point clouds; ‘large’ point clouds indicate high uncertainty). Corresponding color-coding for the anatomical and unconstrained model is missing as these models are not probabilistic. The video is recorded with a frame rate of 200 Hz and slowed down by a factor of 5.

Supplementary Video 5 Same as Supplementary Video 4 but from a different camera view.

Supplementary Video 6 Same as Supplementary Video 4 but data correspond to recordings from a medium-sized animal.

Supplementary Video 7 Same as Supplementary Video 6 but from a different camera view.

Supplementary Video 8 Same as Supplementary Video 4 but data correspond to recordings from a large animal.

Supplementary Video 9 Same as Supplementary Video 8 but from a different camera view.

Supplementary Video 10 Example video from the gait dataset (top) as well as reconstructed skeletal poses projected onto the *x–y*, *x–z* and *y–z* plane (bottom) when the full model is used. For the *x–y*, *x–z* and *y–z* plane view, the first joint of the skeleton graph (the animal’s snout), is fixed with *x* and *y* coordinates equal to 0. Display for uncertainty values of joint and bone positions as in Supplementary Video 4. The video is recorded with a frame rate of 100 Hz and slowed down by a factor of 5.

Supplementary Video 11 Same as Supplementary Video 10 but data correspond to a different behavioral sequence.

Supplementary Video 12 Same as Supplementary Video 10 but data correspond to a different behavioral sequence.

Supplementary Video 13 Example video of gap-crossing showing waiting behavior. Recorded data (left) and reconstructed skeletal poses projected onto the *x–y*, *x–z* and *y–z* plane (right) when the full model is used. For the latter, the first joint of the skeleton graph (the animal’s snout), is fixed with *x* and *y* coordinates equal to 0. Display for uncertainty values of joint and bone positions as in Supplementary Video 4. The video is recorded with a frame rate of 200 Hz and slowed down by a factor of 5.

Supplementary Video 14 Same as Supplementary Video 13 but data correspond to gap-crossing showing direct crossing without waiting.

Supplementary Video 15 Same as Supplementary Video 13 but data correspond to gap-crossing showing reaching behavior.

Supplementary Video 16 Same as Supplementary Video 13 but data correspond to gap-crossing showing reaching behavior.

## Data Availability

The data present in all figures as well as compressed video files are available in the related Dryad repository at 10.5061/dryad.g4f4qrfsw under a CC0 1.0 Universal (CC0 1.0) public domain dedication.

## Code availability

Code for performing pose reconstructions is publicly available on GitHub at https://github.com/bbo-lab/ACM under a LGPL 2.1 license.
