# Supplementary material for: Estimation of skeletal kinematics in freely moving rodents
Source: Nat Methods. 2022 Oct 17;19(11):1500–9. doi: 10.1038/s41592-022-01634-9 (PMC9636019; doi:10.1038/s41592-022-01634-9)
Supplement: Supplementary file 1 — Supplementary Figs. 1–12, Supplementary Text and Supplementary Tables 1–3. [file 41592_2022_1634_MOESM1_ESM.pdf]

nature methods

Article https://doi.org/10.1038/s41592-022-01634-9

Estimation of skeletal kinematics in freely moving rodents

In the format provided by the authors and unedited

Supplementary text

Contents

1. Parameterizing rotations **4**

2. Camera calibration **5**
2.1. Pinhole camera model 5
2.2. Calibration of multiple cameras 6

3. Skeleton model **8**
3.1. Modifying the skeleton model to obtain new poses 8
3.2. Inferring bone lengths and surface marker positions 11
3.3. Scaling of input and output variables 13
3.4. Enforcing body symmetry 15

4. Probabilistic pose estimation **17**
4.1. Using a state space model to describe behavioral time series' 17
4.2. Theory of the expectation-maximization algorithm 20
4.3. The unscented transform 26
4.4. Expectation step 28
4.4.1. The unscented Kalman filter 29
4.4.2. The unscented RTS smoother 31
4.4.3. Enforcing anatomical constraints 32
4.5. Maximization step 34
4.5.1. Obtaining new model parameters by maximizing the evidence lower bound . . 34
4.6. Convergence of the expectation-maximization algorithm 42
4.7. Implementation of the expectation-maximization algorithm 44

5. Training deep neural networks to detect 2D locations of surface markers **46**

6. Box constraints for surface marker positions based on body symmetry **50**

A. Evaluating expected values of log-transformed normal distributions **52**

B. Derivatives **53**

1. Parameterizing rotations

We choose to parameterize rotations with Rodrigues vectors as they are well suited for the description of bone rotations with three rotational degrees of freedom [11]. A Rodrigues vector r is formed by combining the axis of rotation $\omega \in \mathbb{R}^3$ and the rotation angle $\theta \in \mathbb{R}$:

$$r = \theta\omega = \theta(\omega_1, \omega_2, \omega_3)^T \tag{1}$$

where $||\omega|| = 1$. To calculate the associated rotation matrix R from a given Rodrigues vector r we can use the following function:

$$\mathrm{f}_{\mathrm{r}\to\mathrm{R}}(r) = I + \hat{\omega}\sin(\theta) + \hat{\omega}^2(1 - \cos(\theta)) = \begin{pmatrix} R_{11} & R_{12} & R_{13} \\ R_{21} & R_{22} & R_{23} \\ R_{31} & R_{32} & R_{33} \end{pmatrix} = R \tag{2}$$

where $I \in \mathbb{R}^{3\times3}$ is the identity matrix and $\hat{\omega} \in \mathbb{R}^{3\times3}$ is given by:

$$\hat{\omega} = \begin{pmatrix} 0 & -\omega_3 & \omega_2 \\ \omega_3 & 0 & -\omega_1 \\ -\omega_2 & \omega_1 & 0 \end{pmatrix}. \tag{3}$$

2. Camera calibration

2.1. Pinhole camera model

To project an arbitrary three-dimensional joint or surface marker location $m_{\mathrm{3D}} \in \mathbb{R}^3$ onto a camera sensor to obtain the corresponding two-dimensional data point $m_{\mathrm{2D}} \in \mathbb{R}^2$, we are using a pinhole camera model [6], which gives the following relationship between the two:

$$\mathrm{f}_{\mathrm{3D}\to\mathrm{2D}}\left(m_{\mathrm{3D}}, \tilde{r}, \tilde{t}, \tilde{k}, \tilde{A}\right) = \tilde{A}\,\mathrm{f}_{\mathrm{distort}}\left(\mathrm{f}_{\mathrm{r}\to\mathrm{R}}(\tilde{r})\,m_{\mathrm{3D}} + \tilde{t}, \tilde{k}\right) = m_{\mathrm{2D}} \tag{4}$$

where $\tilde{r} \in \mathbb{R}^3$ is the Rodrigues vector and $\tilde{t} \in \mathbb{R}^3$ the translation vector of the respective camera, such that the expression $\mathrm{f}_{\mathrm{r}\to\mathrm{R}}(\tilde{r})\,m_{\mathrm{3D}} + \tilde{t}$ maps m_{3D} from the world coordinate system into the coordinate system of the camera. Given the camera's distortion vector $\tilde{k} \in \mathbb{R}^2$, the function $\mathrm{f}_{\mathrm{distort}}$ applies radial distortions according to

$$\mathrm{f}_{\mathrm{distort}}\left(y, \tilde{k}\right) = \begin{pmatrix} \frac{y_1}{y_3}\left(1 + \tilde{k}_1\tilde{c} + \tilde{k}_2\tilde{c}^2\right) \\ \frac{y_2}{y_3}\left(1 + \tilde{k}_1\tilde{c} + \tilde{k}_2\tilde{c}^2\right) \\ 1 \end{pmatrix} \tag{5}$$

with $y = (y_1, y_2, y_3)^T$ and $\tilde{c} = \left(\frac{y_1}{y_3}\right)^2 + \left(\frac{y_2}{y_3}\right)^2$. The final mapping onto the two-dimensional camera sensor is done using the camera matrix $\tilde{A} \in \mathbb{R}^{2\times3}$ given by

$$\tilde{A} = \begin{pmatrix} \tilde{A}_{11} & 0 & \tilde{A}_{13} \\ 0 & \tilde{A}_{22} & \tilde{A}_{23} \end{pmatrix} \tag{6}$$

where $\tilde{A}_{11}$ and $\tilde{A}_{22}$ are the focal lengths and $\tilde{A}_{13}$ and $\tilde{A}_{23}$ are the x- and y-location of the camera's optical center.

2.2. Calibration of multiple cameras

Given a multi-camera setup with several cameras and overlapping fields of view, we need to infer the initially unknown location and camera parameters of every individual camera in the setup as this allows us to predict where a three-dimensional point in space will be visible on each camera sensor.

This can be achieved by generating a sequence of images showing an object whose physical structure and dimensions are known to us. Hereby, the images are taken synchronously in all cameras, such that the spatial location and orientation of the shown object is identical for a given set of images at a certain time point. For this purpose checkerboards are suited objects as edges of individual tiles can be detected automatically in recorded image frames and the description of their spatial structure requires only a single parameter, i.e. the length of a quadratic tile. Given a multi-camera setup with n_{cam} cameras and n_{time} time points at which we used each camera to record images, which show a checkerboard that has a total of n_{edge} detectable edges, we can calibrate the setup by minimizing a respective objective function via gradient decent optimization using the Trust Region Reflective algorithm [2]:

$$\underset{\substack{\tilde{r}_i,\tilde{t}_i,\tilde{k}_i,\tilde{A}_i,\hat{r}_\tau,\hat{t}_\tau \\ \forall i\in\{1,...,n_{\text{cam}}\} \\ \forall \tau\in\{1,...,n_{\text{time}}\}}}{\arg\min} \sum_{\tau=1}^{n_{\text{time}}} \sum_{i=1}^{n_{\text{cam}}} \sum_{j=1}^{n_{\text{edge}}} \delta_{\tau ij} \left|\left| m_{\tau ij} - \mathrm{f}_{\text{3D}\rightarrow\text{2D}} \left(\mathrm{f}_{\text{r}\rightarrow\text{R}} \left(\hat{r}_\tau \right) \hat{m}_j + \hat{t}_\tau, \tilde{r}_i, \tilde{t}_i, \tilde{k}_i, \tilde{A}_i \right) \right|\right|^2 \tag{7}$$

where $\tilde{r}_i$ is the Rodrigues vector, $\tilde{t}_i$ is the translation vector, $\tilde{k}_i$ is the distortion vector and $\tilde{A}_i$ is the camera matrix of camera i. The Rodrigues vector $\hat{r}_\tau$ and the translation vector $\hat{t}_\tau$ encode the orientation and translation of the checkerboard at time point τ. Since the checkerboard is a planar object each edge j is given by a three-dimensional point $\hat{m}_j = c_{\text{tile}}(x_j, y_j, 0)^T$ with the known length of a single tile c_{tile} and $x_j \in \mathbb{N}$ as well as $y_j \in \mathbb{N}$. Furthermore, the two-dimensional edge j in camera i at time point τ is denoted as $m_{\tau ij} \in \mathbb{R}^2$ and the delta function $\delta_{\tau ij}$ indicates whether this edge is detected successfully, i.e. $\delta_{\tau ij} = 1$, or not, i.e. $\delta_{\tau ij} = 0$.

3. Skeleton model

3.1. Modifying the skeleton model to obtain new poses

Given a three-dimensional skeleton model, we need to adjust joint locations by rotating each bone of the model, such that resulting three-dimensional positions of rigidly attached surface markers match the respective two-dimensional locations in our video data. Assuming our skeleton model has a total of n_{bone} bones and n_{marker} surface markers, we want to generate the three-dimensional locations of the joints $p \in \mathbb{R}^{n_{\text{bone}}+1\times 3}$ and surface markers $m \in \mathbb{R}^{n_{\text{marker}}\times 3}$, which can be obtained according to Algorithm 1.

Algorithm 1

function $\mathrm{f}_{\text{pose}}(t, r, l, v)$
  for $j \in \{1, ..., n_{\text{bone}}\}$ **do**
    $R_j \leftarrow I$ ▷ Initialize each bone rotation R_j
  for $i \in \{1, ..., n_{\text{bone}}\}$ **do**
    for $j \in \{1, ..., n_{\text{bone}}\}$ **do**
      if j_1 is child of i_0 **then** ▷ Check if rotation of bone i affects end joint j_1
        $R_j \leftarrow \mathrm{f}_{\text{r}\rightarrow\text{R}} \left(r_i \right)^T R_j$ ▷ Update rotation of bone j
  for $j \in \{1, ..., n_{\text{bone}}\}$ **do**
    $R_j \leftarrow {R_j}^T \bar{R}_j$ ▷ Apply bone rotation R_j to resting pose $\bar{R}_j$
  $p_{1_0} \leftarrow t$ ▷ Initialize root joint location p_{1_0}
  for $j \in \{1, ..., n_{\text{bone}}\}$ **do**
    $p_{j_1} \leftarrow p_{j_0} + \left(R_{j_{13}}, R_{j_{23}}, R_{j_{33}} \right)^T l_j$ ▷ Calculate end joint location p_{j_1} of bone j
    for $k \in \{1, ..., n_{\text{marker}}\}$ **do**
      if j_1 is connected to k **then** ▷ Check if end joint j_1 is connected to marker k
        $m_k \leftarrow p_{j_1} + R_j v_k$ ▷ Calculate absolute marker location m_k
  return m

Here, it is assumed that the set $\{1, ..., n_{\text{bone}}\}$ is sorted, such that one iterates through the skeleton graph beginning with the bone whose start joint is the root joint 1_0 and then proceed with the bones further down the skeleton graph. Thus, it is always guaranteed that for $j > i$, the start joint i_0 of bone i is never a child of the start joint j_0 of bone j. It is also assumed that the bone coordinate systems of the skeleton model are constructed such that their z-directions encode the directions in which the respective bones are pointing. Furthermore, the global translation vector $t \in \mathbb{R}^3$ corresponds to the three-dimensional location of the skeleton's root joint, the rows of the tensor $r \in \mathbb{R}^{n_{\text{bone}} \times 3}$ contain Rodrigues vectors encoding the bone rotations, the vector $l \in \mathbb{R}^{n_{\text{bone}}}$ contains the bone lengths and the rows of the tensor $v \in \mathbb{R}^{n_{\text{marker}} \times 3}$ contain the relative maker locations, i.e. the locations of the markers when the position of the attached joints are assumed to be the origin. The resting pose $\bar{R} \in \mathbb{R}^{n_{\text{bone}} \times 3 \times 3}$ of the animal describes the orientation of the bones when no additional rotations are applied, i.e. $r_i = (0, 0, 0)^T \ \forall \ i \in \{1, ..., n_{\text{bone}}\}$. Here, the frequent usage of the transpose operation allows to first rotate bones, which are the closest to the leaf joints of the skeleton graph [4]. This has the advantage that we can enforce constraints on bone rotations with reference to a global coordinate system that corresponds to the three main axes of the animal's body. Assume we only model a single front limb where we only have rotations around the shoulder, elbow and wrist, i.e. R_{shoulder}, R_{elbow} and R_{wrist}, and would like to obtain the new orientation R_{new} of the bone whose start joint is identical to the animal's wrist given its resting pose $\bar{R}_{\text{wrist}}$ while iterating through the skeleton graph starting from the root joint, i.e. the shoulder. Then we can obtain R_{new} according to

$$R_{\text{new}} = \left({R_{\text{wrist}}}^T {R_{\text{elbow}}}^T {R_{\text{shoulder}}}^T \right)^T \bar{R}_{\text{wrist}} = R_{\text{shoulder}} R_{\text{elbow}} R_{\text{wrist}} \bar{R}_{\text{wrist}} \tag{8}$$

Thus, we can iterate through the skeleton graph from the root to the leaf joints but actually apply the respective bone rotations in the reversed order.

3.2. Inferring bone lengths and surface marker positions

Reconstructing poses for n_{time} time points can be archived equivalently to the calibration of a multi-camera setup as discussed in Section 2.2, i.e. we need to minimize a respective objective function via gradient decent optimization using the L-BFGS-B algorithm [3]:

$$\underset{\substack{t_\tau, r_\tau, l, v \\ \forall \tau \in \{1, ..., n_{\text{time}}\}}}{\arg\min} \sum_{\tau=1}^{n_{\text{time}}} \sum_{i=1}^{n_{\text{cam}}} \sum_{j=1}^{n_{\text{marker}}} \delta_{\tau ij} ||m_{\tau ij} - \hat{m}_{\tau ij}||^2 \tag{9}$$

where $m_{\tau ij}$ is the two-dimensional location of marker j in camera i at time point τ and $\delta_{\tau ij}$ indicates whether this marker location was successfully detected, i.e. $\delta_{\tau ij} = 1$, or not, i.e. $\delta_{\tau ij} = 0$. The corresponding projected two-dimensional marker location $\hat{m}_{\tau ij}$ can be obtained by propagating the absolute marker positions calculated via Algorithm 1 through the projection function $\text{f}_{\text{3D}\to\text{2D}}$:

$$\hat{m}_{\tau ij} = \text{f}_{\text{3D}\to\text{2D}} \left(\text{f}_{\text{pose}} \left(t_\tau, r_\tau, l, v \right)_j, \tilde{r}_i, \tilde{t}_i, \tilde{k}_i, \tilde{A}_i \right) \tag{10}$$

where $t_\tau \in \mathbb{R}^3$ and $r_\tau \in \mathbb{R}^{n_{\text{bone}} \times 3}$ denote the translation vector and the bone rotations at time point τ. Note how there is a set of pose-encoding parameters t_τ and r_τ for each time point τ whereas the bone lengths l and the relative surface marker positions v, which encode the animal's skeletal structure and configuration, are shared across all time points. Thus, if we provide enough time points where the animal is visible in many different poses, which ideally cover the entire spectrum of the animal's behavioral space, we can not only reconstruct the pose of the animal for the given time points but are also able to learn the structure of the animal's skeleton, by inferring the unknown parameters l and v.

3.3. Scaling of input and output variables

In general, we always scale the translation vector t and the bone rotations r as well as the resulting two-dimensional marker locations $\hat{m}$, such that all of them roughly lie within the same range, i.e.

$[-1, 1]$. Particularly, we define the normalization constants $c_t = 50$ cm and $c_r = \frac{\pi}{2}$ rad as well as $c_1 = 640$ px and $c_2 = 512$ px, which we use to normalize r and t as well as $\hat{m}$. The choice for c_t was based on the dimensions of the largest arena we used in our experiments, where the maximum distance to an arena's edge from the origin of the world coordinate system, located at the center of the arena, was around 50 cm. The choice for c_r was based on the maximum bone rotation of the naively constrained spine and tail joints in our skeleton model, which was equal to $\frac{\pi}{2}$ rad. The choice for c_1 and c_2 were based on the sensor sizes of the cameras we used in our experiments, which were all equal to 1280×1024 px^2. Using the normalization constants we obtain the normalized translation vector $t^* = \frac{t}{c_t}$ and the normalized bone rotations $r^* = \frac{r}{c_r}$ as well as the normalized two-dimensional marker locations

$$\hat{m}^* = \begin{pmatrix} \hat{m}_1^* \\ \hat{m}_2^* \end{pmatrix} = \begin{pmatrix} \frac{\hat{m}_1}{c_1} - 1 \\ \frac{\hat{m}_2}{c_2} - 1 \end{pmatrix} \tag{11}$$

for a single two dimensional marker location $\hat{m} \in \mathbb{R}^2$, such that $\hat{m}_1$ represents its x- and $\hat{m}_2$ its y-coordinate. These normalized variables were used instead of their non-normalized counterparts in all depicted optimization and pose reconstruction steps.

3.4. Enforcing body symmetry

To improve the inference of bone lengths and surface marker positions we took advantage of the symmetric properties of an animal's body, i.e. for every left-sided limb there exists a corresponding limb on the right side. Furthermore, we also placed the surface markers onto the animal's fur, such that the marker-pattern itself was symmetrical, e.g. for a marker that was placed to a position close to the left hip joint there was a corresponding marker on the right side of the animal. By incorporating this knowledge into Algorithm 1 we reduced the number of free parameters, i.e. we only optimized the reduced bone lengths $l^* \in \mathbb{R}^{n^*_{\text{bone}}}$ and relative marker positions $v^* \in \mathbb{R}^{n^*_{\text{marker}} \times 3}$, where n^*_{bone} is the number of asymmetrical bones, i.e. bones along the head, spine and tail, plus the number of limb bones on the animal's left side and, equivalently, n^*_{marker} denotes the number of the asymmetrical and left-sided markers. The excluded right-sided limb bones were then enforced to have the same lengths as the corresponding limb bones on the left side. Additionally, we also applied this concept for the relative marker locations by mirroring the x-component of the left-sided markers at the yz-plane to obtain the relative marker locations of the markers on the right side. To implement this we defined Algorithm 2, which maps the reduced bone lengths l^* to the original parameter l.

Algorithm 2

function $\mathrm{f}_{l^* \to l}(l^*)$
  $c \leftarrow 1$ ▷ Initialize counter c for right-sided bones
  for $i \in \{1, ..., n^*_{\text{bone}}\}$ **do**
    $l_i \leftarrow l^*_i$ ▷ Set asymmetric/left-sided bone length l_i
    if i is left-sided bone **then** ▷ Check if bone i is on the left side
      $l_{n^*_{\text{bone}}+c} \leftarrow l^*_i$ ▷ Set right-sided bone length $l_{n^*_{\text{bone}}+c}$
      $c \leftarrow c + 1$ ▷ Increase counter c for right-sided bones
  return l

Equivalently, we also defined the corresponding Algorithm 3, which maps the reduced relative marker positions v^* to their original counterpart v.

Algorithm 3

function $\mathrm{f}_{v^* \to v}(v^*)$
  $c \leftarrow 1$ ▷ Initialize counter c for right-sided markers
  for $j \in \{1, ..., n^*_{\mathrm{marker}}\}$ **do**
    $v_j \leftarrow v^*_j$ ▷ Set asymmetric/left-sided rel. marker position v_j
    if j is left-sided marker **then** ▷ Check if marker j is on the left side
      $v_{n^*_{\mathrm{marker}}+c} \leftarrow \left(-v^*_{j\,1}, v^*_{j\,2}, v^*_{j\,3}\right)^T$ ▷ Set right-sided rel. marker position $v_{n^*_{\mathrm{marker}}+c}$
      $c \leftarrow c+1$ ▷ Increase counter c for right-sided markers
  return v

To learn the underlying three-dimensional skeleton model while also enforcing body symmetry, we then redefined $\hat{m}_{\tau ij}$ from equation 10 as follows:

$$\hat{m}_{\tau ij} = \mathrm{f}_{\mathrm{3D}\to\mathrm{2D}}\left(\mathrm{f}_{\mathrm{pose}}\left(t_\tau, r_\tau, \mathrm{f}_{\mathrm{l}^*\to\mathrm{l}}\left(l^*\right), \mathrm{f}_{\mathrm{v}^*\to\mathrm{v}}\left(v^*\right)\right)_j, \tilde{r}_i, \tilde{t}_i, \tilde{k}_i, \tilde{A}_i\right) \tag{12}$$

and minimized equation 9 with respect to the parameters l^* and v^* instead of l and v.

4. Probabilistic pose estimation

4.1. Using a state space model to describe behavioral time series'

To allow for probabilistic pose reconstruction of entire behavioral sequences of length T, which ensures that poses of consecutive time points are similar to each other, we deploy a state space model, given by a transition and an emission equation

$$z_t = z_{t-1} + \epsilon_z \tag{13}$$

$$x_t = \mathrm{g}\left(z_t\right) + \epsilon_x \tag{14}$$

where at time point $t \in \{1, ..., T\}$ the state variable $z_t \in \mathbb{R}^{n_z}$ encodes the position of the animal as well as the bone rotations and the measurement variable $x_t \in \mathbb{R}^{n_x}$ represents the two-dimensional surface marker locations in all cameras given by a trained neural network. Thus, the state variable z_t contains the global translation vector t as well as the pose-encoding tensor r for time point t and the measurement variable x_t is a constant quantity given for all time points t. The function g, given by Algorithm 4, computes the noise-free measurements of the two-dimensional surface marker locations x^*_t given the state variable z_t. At this point the bone lengths l and relative maker locations v are already inferred and therefore given. The same applies to the Rodrigues vector $\tilde{r}_i$, the translation vector $\tilde{t}_i$, the distortion vector $\tilde{k}_i$ and the camera matrix $\tilde{A}_i$ of camera i, which we obtained from calibrating the multi-camera setup. The normalization constants c_t, c_r as well as c_1 and c_2 are the same as in Section 3.3. The probabilistic nature of the model is given by incorporating the two normally distributed random variables $\epsilon_z \sim \mathcal{N}\left(0, V_z\right)$ and $\epsilon_x \sim \mathcal{N}\left(0, V_x\right)$, simulating small pose changes over time and measurement noise, as well as the initial state $z_0 \sim \mathcal{N}\left(\mu_0, V_0\right)$, which is also assumed to be a normally distributed random variable. Thus, the state space model is entirely described by the model parameters $\Theta = \{\mu_0, V_0, V_z, V_x\}$. This allows for inferring a set of expected state variables $z = \{z_1, ..., z_T\}$ given our measurements $x = \{x_1, ..., x_T\}$ in case we have a good estimate for the model parameters Θ. Alternatively, we are also able to calculate a set of model parameters Θ, which, given an estimate for the state variables z, maximizes a lower bound of the model's evidence, i.e. the evidence lower bound (ELBO). The former is equivalent to the expectation step (E-step) of the expectation-maximization (EM) algorithm, which can be performed by applying the unscented Rauch-Tung-Striebel (RTS) smoother, whereas the latter is identical to the algorithm's maximization step (M-step), in which new model parameters are calculated in closed form to maximize the ELBO [8].

Algorithm 4

function $\mathrm{g}(z_t)$
  $t \leftarrow c_t(z_{t1}, z_{t2}, z_{t3})^T$ ▷ Obtain global translation t
  for $i \in \{1, .., n_{\text{bone}}\}$ **do**
    $r_i \leftarrow c_r(z_{t3i+1}, z_{t3i+2}, z_{t3i+3})^T$ ▷ Obtain bone rotation r_i
  $m_{\text{3D}} \leftarrow \mathrm{f}_{\text{pose}}(t, r, l, v)$ ▷ Obtain 3D marker locations given l and v
  for $i \in \{1, .., n_{\text{cam}}\}$ **do**
    for $j \in \{1, .., n_{\text{marker}}\}$ **do**
      $m_{\text{2D}} \leftarrow \mathrm{f}_{\text{3D}\to\text{2D}}\left(m_{\text{3D}j}, \tilde{r}_i, \tilde{t}_i, \tilde{k}_i, \tilde{A}_i\right)$ ▷ Obtain 2D marker locations given $\tilde{r}$, $\tilde{t}$, $\tilde{k}$ and $\tilde{A}$
      $m^*_{n_{\text{marker}}(i-1)+j} \leftarrow \left(\frac{m_{\text{2D}_1}}{c_1} - 1, \frac{m_{\text{2D}_2}}{c_2} - 1\right)^T$ ▷ Normalize x- and y-coordinates
  $x^*_t \leftarrow \mathrm{cat}\left(m^*_1, m^*_2, ..., m^*_{n_{\text{cam}} n_{\text{marker}}}\right)$ ▷ Obtain noise-free x^*_t via concatenation
  return x^*_t

4.2. Theory of the expectation-maximization algorithm

While the EM algorithm was first introduced by Dempster et al. [5], we follow the concepts and notations stated by Bishop [1] and Murphy [10]. To derive a formulation of the ELBO we first note that the model's joint distribution $\mathrm{p}(x, z)$ is equal to the product of the model's likelihood $\mathrm{p}(x|z)$ and prior $\mathrm{p}(z)$:

$$\mathrm{p}(x, z) = \mathrm{p}(x|z)\,\mathrm{p}(z)\,. \tag{15}$$

Additionally, we also note that the mutual dependency of the model's marginal likelihood $\mathrm{p}(x)$, posterior $\mathrm{p}(z|x)$, likelihood $\mathrm{p}(x|z)$ and prior $\mathrm{p}(z)$ is given by Bayes' theorem:

$$\mathrm{p}(z|x)\,\mathrm{p}(x) = \mathrm{p}(x|z)\,\mathrm{p}(z)\,. \tag{16}$$

We now define an arbitrary probability density function $\mathrm{q}(z)$ over our state variables z, for which we know the following statement is true by definition:

$$\int \mathrm{q}(z)\,dz = 1. \tag{17}$$

Multiplying equation 17 with an arbitrary constant c yields:

$$c \int \mathrm{q}(z)\,dz = \int c\,\mathrm{q}(z)\,dz = c. \tag{18}$$

We can now replace the constant c with a function independent from the state variables z without loss of generality. If we choose this function to be the model's marginal log-likelihood $\ln \mathrm{p}(x)$, we obtain:

$$\int \mathrm{q}(z) \ln \mathrm{p}(x)\,dz = \ln \mathrm{p}(x) \tag{19}$$

and note that, due to equation 17 and 18 respectively, the marginal log-likelihood $\ln \mathrm{p}(x)$ is actually independent of the probability density function $\mathrm{q}(z)$. Next, we can use equation 15 and 16 to derive a relationship between the marginal log-likelihood $\ln \mathrm{p}(x)$, the Kullback–Leibler (KL) divergence

$\mathrm{KL}\left(q||p\right)$ and the ELBO $\mathcal{L}$, starting from equation 19:

$$
\begin{aligned}
\ln \mathrm{p}\left(x\right) &= \int \mathrm{q}\left(z\right) \ln \mathrm{p}\left(x\right) dz && (20)\\
&= \int \mathrm{q}\left(z\right) \ln \frac{\mathrm{p}\left(z|x\right) \mathrm{p}\left(x\right)}{\mathrm{p}\left(z|x\right)} dz && (21)\\
&= \int \mathrm{q}\left(z\right) \ln \frac{\mathrm{p}\left(x|z\right) \mathrm{p}\left(z\right)}{\mathrm{p}\left(z|x\right)} dz && (22)\\
&= \int \mathrm{q}\left(z\right) \ln \frac{\mathrm{p}\left(x,z\right)}{\mathrm{p}\left(z|x\right)} dz && (23)\\
&= \int \mathrm{q}\left(z\right) \ln \frac{\mathrm{p}\left(x,z\right) \mathrm{q}\left(z\right)}{\mathrm{p}\left(z|x\right) \mathrm{q}\left(z\right)} dz && (24)\\
&= \int \mathrm{q}\left(z\right) \left(\ln \frac{\mathrm{p}\left(x,z\right)}{\mathrm{q}\left(z\right)} - \ln \frac{\mathrm{p}\left(z|x\right)}{\mathrm{q}\left(z\right)} \right) dz && (25)\\
&= \int \mathrm{q}\left(z\right) \ln \frac{\mathrm{p}\left(x,z\right)}{\mathrm{q}\left(z\right)} dz - \int \mathrm{q}\left(z\right) \ln \frac{\mathrm{p}\left(z|x\right)}{\mathrm{q}\left(z\right)} dz && (26)\\
&= \mathcal{L} + \mathrm{KL}\left(q||p\right) && (27)
\end{aligned}
$$

with $\mathcal{L} = \int \mathrm{q}\left(z\right) \ln \frac{\mathrm{p}(x,z)}{\mathrm{q}(z)} dz$ and $\mathrm{KL}\left(q||p\right) = -\int \mathrm{q}\left(z\right) \ln \frac{\mathrm{p}(z|x)}{\mathrm{q}(z)} dz$. The KL divergence is a distance measure between the probability density functions q and p and as such always larger or equal to zero:

$$\mathrm{KL}\left(q||p\right) \geq 0 \tag{28}$$

with equality $\mathrm{KL}\left(q||p\right) = 0$ if $q = p$. When we add the ELBO $\mathcal{L}$ to equation 28 and combine the result with the derived definition of $\ln \mathrm{p}\left(x\right)$, it becomes clear that the ELBO $\mathcal{L}$ is a lower bound of the marginal log-likelihood:

$$\ln \mathrm{p}\left(x\right) = \mathcal{L} + \mathrm{KL}\left(q||p\right) \geq \mathcal{L}. \tag{29}$$

If we now acknowledge that we also require the model parameters Θ to compute the above quantities, i.e.

$$
\begin{aligned}
\ln \mathrm{p}\left(x|\Theta\right) &= \mathcal{L}\left(\mathrm{q}, \Theta\right) + \mathrm{KL}\left(q||p\right) && (30)\\
&= \int \mathrm{q}\left(z\right) \ln \frac{\mathrm{p}\left(x,z|\Theta\right)}{\mathrm{q}\left(z\right)} dz - \int \mathrm{q}\left(z\right) \ln \frac{\mathrm{p}\left(z|x,\Theta\right)}{\mathrm{q}\left(z\right)} dz && (31)\\
&= \left(\int \mathrm{q}\left(z\right) \ln \mathrm{p}\left(x|\Theta\right) dz + \int \mathrm{q}\left(z\right) \ln \frac{\mathrm{p}\left(z|x,\Theta\right)}{\mathrm{q}\left(z\right)} dz \right) - \int \mathrm{q}\left(z\right) \ln \frac{\mathrm{p}\left(z|x,\Theta\right)}{\mathrm{q}\left(z\right)} dz && (32)\\
&\geq \mathcal{L}\left(\mathrm{q}, \Theta\right) && (33)\\
&= \int \mathrm{q}\left(z\right) \ln \mathrm{p}\left(x|\Theta\right) dz - \mathrm{KL}\left(q||p\right), && (34)
\end{aligned}
$$

we can start building an understanding for how the EM algorithm works. In the E-step we are holding Θ constant and maximize $\mathcal{L}\left(\mathrm{q}, \Theta\right)$ with respect to q, i.e. given a current estimate for the model parameters Θ_k we infer the probability density functions of our state variables $\mathrm{p}\left(z|x, \Theta_k\right)$, such that $\mathrm{q}\left(z\right) = \mathrm{p}\left(z|x, \Theta_k\right)$, making the KL divergence $\mathrm{KL}\left(q||p\right)$ become zero, i.e. $\mathrm{KL}\left(q||p\right) = \mathrm{KL}\left(p||p\right) = 0$, and the marginal log-likelihood $\ln \mathrm{p}\left(x|\Theta_k\right)$ become equal to the ELBO $\mathcal{L}\left(q, \Theta\right)$. Here, setting $\mathrm{q}\left(z\right) = \mathrm{p}\left(z|x, \Theta_k\right)$ maximizes the ELBO $\mathcal{L}\left(\mathrm{q}, \Theta\right)$ due to the equality given by equation 34 and the previously mentioned fact that the marginal log-likelihood $\ln \mathrm{p}\left(x\right)$ is actually independent of the probability density function $\mathrm{q}\left(z\right)$. Subsequently, in the M-step we are holding q constant and maximize $\mathcal{L}\left(\mathrm{q}, \Theta\right)$ with respect to Θ in order to obtain a new set of model parameters Θ_{k+1}, leading to an increased marginal log-likelihood $\ln \mathrm{p}\left(x|\Theta_{k+1}\right)$, as the KL divergence becomes greater then zero again, i.e. $\mathrm{KL}\left(q||p\right) \geq 0$

and $\mathcal{L}(\mathrm{q}, \Theta_{k+1}) \geq \mathcal{L}(\mathrm{q}, \Theta_k)$. Thus, the starting point in the M-step is the following:

$$\ln \mathrm{p}(x|\Theta) \geq \mathcal{L}(\mathrm{q}, \Theta) \tag{35}$$
$$= \int \mathrm{p}(z|x, \Theta_k) \ln \frac{\mathrm{p}(x, z|\Theta)}{\mathrm{p}(z|x, \Theta_k)} dz \tag{36}$$
$$= \int \mathrm{p}(z|x, \Theta_k) \ln \mathrm{p}(x, z|\Theta)\, dz - \int \mathrm{p}(z|x, \Theta_k) \ln \mathrm{p}(z|x, \Theta_k)\, dz \tag{37}$$
$$= \mathcal{Q}(\Theta, \Theta_k) - \int \mathrm{p}(z|x, \Theta_k) \ln \mathrm{p}(z|x, \Theta_k)\, dz \tag{38}$$

with $\mathcal{Q}(\Theta, \Theta_k) = \int \mathrm{p}(z|x, \Theta_k) \ln \mathrm{p}(x, z|\Theta)\, dz$. We note that the latter term is independent of Θ and can be omitted since our goal is to optimize the ELBO $\mathcal{L}(\mathrm{q}, \Theta)$ with respect to Θ. Therefore, instead of maximizing the ELBO $\mathcal{L}(\mathrm{q}, \Theta)$ directly, we can just maximize the function $\mathcal{Q}(\Theta, \Theta_k)$. We furthermore notice that $\mathcal{Q}(\Theta, \Theta_k)$ has the form of an expectation value, i.e. we can obtain $\mathcal{Q}(\Theta, \Theta_k)$ by taking the expectation of $\ln \mathrm{p}(x, z|\Theta)$ with respect to z:

$$\mathcal{Q}(\Theta, \Theta_k) = \mathbb{E}[\ln \mathrm{p}(x, z|\Theta)] \tag{39}$$

where $\mathbb{E}[\ln \mathrm{p}(x, z|\Theta)]$ is conditioned on x and Θ_k, i.e. both quantities are given. With this we finally arrive at the essence of what is done during the M-step, i.e. maximizing $\mathcal{Q}(\Theta, \Theta_k)$ with respect to Θ to obtain new model parameters Θ_{k+1}:

$$\Theta_{k+1} = \arg\max_{\Theta} \mathcal{Q}(\Theta, \Theta_k) \tag{40}$$

4.3. The unscented transform

We are required to approximate expectation values to perform the E-step, i.e. when applying the unscented Kalman filter and the unscented RTS smoother (Algorithm 7 and 9), as well as the M-step, i.e. when maximizing $\mathcal{Q}(\Theta, \Theta_k)$ (equation 39), as we can not compute them analytically [8]. These expectation values are of the form:

$$\mathbb{E}[\mathrm{h}(y)] = \int \mathrm{p}(y)\, \mathrm{h}(y)\, dy \tag{41}$$

where h is an arbitrary function and $y \in \mathbb{R}^d$ an arbitrary normally distributed random variable, i.e. $y \sim \mathcal{N}(m, \Sigma)$. We can obtain such approximations using the unscented transform f_{ut}, which was first introduced by Julier et al. [7] and is defined in Algorithm 5. Given the mean m and the covariance Σ, the unscented transform f_{ut} generates so called sigma points $\mathcal{Y} \in \mathbb{R}^{2d+1 \times d}$, whose locations are systematically spread around the mean m based on the covariance Σ:

Algorithm 5

```
function f_ut(m, Σ)
    L ← f_cholesky(Σ)
    Y_1 ← m
    for i ∈ {2, ..., d + 1} do
        Y_i ← m + √(d + λ) L^T_i
    for i ∈ {d + 2, ..., 2d + 1} do
        Y_i ← m − √(d + λ) L^T_i
    return Y
```

Here $\mathrm{f}_{\mathrm{cholesky}}(\Sigma)$ denotes the Cholesky decomposition of matrix Σ, which computes a lower triangular matrix L such that $LL^T = \Sigma$, and λ can be calculated as follows:

$$\lambda = \alpha^2 (d + \kappa) - d \tag{42}$$

where we set the parameters $\alpha = 1$ and $\kappa = 0$ [8], such that $\lambda = 0$. Using the sigma points $\mathcal{Y}$, we can approximate $\mathbb{E}\left[\mathrm{h}\left(y\right)\right]$ as follows:

$$\mathbb{E}\left[\mathrm{h}\left(y\right)\right] \approx \sum_{i=1}^{2d+1} w_i \, \mathrm{h}\left(\mathcal{Y}_i\right) = \sum_{i=1}^{2d+1} w_i \, \mathrm{h}\left(\mathrm{f}_{\mathrm{ut}}\left(m, \Sigma\right)_i\right) \tag{43}$$

with the weights w:

$$w_1 = \frac{\lambda}{d + \lambda} \tag{44}$$

$$w_i = \frac{1}{2\left(d + \lambda\right)} \forall i \in \{2, ..., 2d+1\}. \tag{45}$$

which due to our choice of α and κ simplifies to:

$$w_1 = 0 \tag{46}$$

$$w_i = \frac{1}{2d} \forall i \in \{2, ..., 2d+1\}. \tag{47}$$

4.4. Expectation step

In the E-step we need to infer the probability density function of the latent variable z_t for all time points t of a behavioral sequence, given the set of all measurements x and the model parameters Θ, noted as $\mathrm{p}\left(z_t | x, \Theta\right)$. Since all random variables of the model are assumed to be normally distributed, this property is maintained for the latent variable z_t as well. Therefore, z_t is drawn form a normal distribution with mean μ_t and covariance V_t, i.e. $z_t \sim \mathcal{N}\left(\mu_t, V_t\right)$. By using all measurements x of the sequence for the inference of $\mathrm{p}\left(z_t | x, \Theta\right)$ at time point t, information of the past as well as of the future is processed, which is what the unscented RTS smoother is used for. However, to derive the equations of the smoother we first need to focus on the inference when only information of the past is available, i.e. we want to infer $\mathrm{p}\left(z_t | x_1, ..., x_t, \Theta\right)$ where only measurements until time point t are given, which can be achieved by utilizing the unscented Kalman filter. To avoid confusions, we denote mean values and covariance matrices obtained from the unscented Kalman filter as $\tilde{\mu}_t$ and $\tilde{V}_t$, whereas those calculated via the unscented RTS smoother are denoted as $\hat{\mu}_t$ and $\hat{V}_t$.

4.4.1. The unscented Kalman filter

The unscented Kalman filter is an iterative algorithm, which calculates the filtered values for the mean $\tilde{\mu}_t$ and covariance $\tilde{V}_t$ at a time point t, based on the filter output for these values $\tilde{\mu}_{t-1}$ and $\tilde{V}_{t-1}$ at the previous time point $t-1$ as well as the measurement variable x_t for time point t. The inference scheme for obtaining $\mathrm{p}\left(z_t | x_1, ..., x_{t-1}, \Theta\right)$ is given by Algorithm 6 and 7 [12, 13]:

Algorithm 6

function $\mathrm{f}_{\mathrm{ukf}_0}(\tilde{\mu}_{t-1}, \tilde{V}_{t-1}, V_z, V_x)$
 $\mathcal{Z} \leftarrow \mathrm{f}_{\mathrm{ut}}\left(\tilde{\mu}_{t-1}, \tilde{V}_{t-1}\right)$ ▷ Form sigma points $\mathcal{Z}$
 $\bar{z} \leftarrow \sum_{i=1}^{2n_z+1} w_i \mathcal{Z}_i$ ▷ Compute predicted mean $\bar{z}$
 $P \leftarrow V_z + \sum_{i=1}^{2n_z+1} w_i(\mathcal{Z}_i - \bar{z})(\mathcal{Z}_i - \bar{z})^T$ ▷ Compute predicted covariance P
 $\mathcal{Z} \leftarrow \mathrm{f}_{\mathrm{ut}}(\bar{z}, P)$ ▷ Form sigma points $\mathcal{Z}$
 $\mathcal{X} \leftarrow \mathrm{g}(\mathcal{Z})$ ▷ Propagate sigma points through emission function g
 $\bar{x} \leftarrow \sum_{i=1}^{2n_z+1} w_i \mathcal{X}_i$ ▷ Compute predicted mean $\bar{x}$
 $S \leftarrow V_x + \sum_{i=1}^{2n_z+1} w_i(\mathcal{X}_i - \bar{x})(\mathcal{X}_i - \bar{x})^T$ ▷ Compute predicted covariance S
 for $i \in \{1, ..., n_x\}$ **do**
  if x_{ti} is missing measurement **then**
   for $j \in \{1, ..., n_x\}$ **do**
    $S_{ij} \leftarrow 0$ ▷ Set rows of missing measurements to 0
    $S_{ji} \leftarrow 0$ ▷ Set columns of missing measurements to 0
   $S_{ii} \leftarrow 1$ ▷ Set diagonal entries to 1 to allow computing S^{-1}
 $C \leftarrow \sum_{i=1}^{2n_z+1} w_i(\mathcal{Z}_i - \bar{z})(\mathcal{X}_i - \bar{x})^T$ ▷ Compute cross-covariance C
 for $i \in \{1, ..., n_x\}$ **do**
  if x_{ti} is missing measurement **then**
   for $j \in \{1, ..., n_z\}$ **do**
    $C_{ji} \leftarrow 0$ ▷ Set columns of missing measurements to 0
 $K \leftarrow CS^{-1}$ ▷ Compute filter gain K
 $\bar{x} \leftarrow x_t - \bar{x}$
 for $i \in \{1, ..., n_x\}$ **do**
  if x_{ti} is missing measurement **then**
   $\bar{x}_i \leftarrow 0$ ▷ Set entries of missing measurements to 0
 $\tilde{\mu}_t \leftarrow \bar{z} + K\bar{x}$ ▷ Compute filtered mean $\tilde{\mu}_t$
 $\tilde{V}_t \leftarrow P - KC^T$ ▷ Compute filtered covariance $\tilde{V}_t$
 return $\tilde{\mu}_t, \tilde{V}_t$

To obtain values for filtered means $\tilde{\mu} = \{\tilde{\mu}_0, ..., \tilde{\mu}_T\}$ and covariances $\tilde{V} = \{\tilde{V}_0, ..., \tilde{V}_T\}$ for all time points one needs to iterate through the entire behavioral sequence:

Algorithm 7

function $\mathrm{f}_{\mathrm{ukf}}(\mu_0, V_0, V_z, V_x)$
 $\tilde{\mu}_0 \leftarrow \mu_0$
 $\tilde{V}_0 \leftarrow V_0$
 for $t \in \{1, ..., T\}$ **do**
  $\tilde{\mu}_t, \tilde{V}_t \leftarrow \mathrm{f}_{ukf_0}\left(\tilde{\mu}_{t-1}, \tilde{V}_{t-1}, V_z, V_x\right)$
 return $\tilde{\mu}, \tilde{V}$

4.4.2. The unscented RTS smoother

The unscented RTS smoother is also an iterative algorithm, which calculates the smoothed values for the mean $\hat{\mu}_t$ and covariance $\hat{V}_t$ at a time point t, based on the smoother output for these values $\hat{\mu}_{t+1}$ and $\hat{V}_{t+1}$ at the next time point $t+1$ as well as the corresponding output from the unscented Kalman filter $\tilde{\mu}_t$ and $\tilde{V}_t$ for time point t. The inference scheme for obtaining $\mathrm{p}(z_t|x, \Theta)$ is given by Algorithm 8 and 9 [13]:

Algorithm 8

function $\mathrm{f}_{\mathrm{uks}_0}(\tilde{\mu}_t, \tilde{V}_t, \hat{\mu}_{t+1}, \hat{V}_{t+1}, V_z)$
  $\mathcal{Z} \leftarrow \mathrm{f}_{\mathrm{ut}}\left(\tilde{\mu}_t, \tilde{V}_t\right)$ ▷ Form sigma points $\mathcal{Z}$
  $\bar{z} \leftarrow \sum_{i=1}^{2n_z+1} w_i \mathcal{Z}_i$ ▷ Compute predicted mean $\bar{z}$
  $P \leftarrow V_z + \sum_{i=1}^{2n_z+1} w_i (\mathcal{Z}_i - \bar{z})(\mathcal{Z}_i - \bar{z})^T$ ▷ Compute predicted covariance P
  $D \leftarrow \sum_{i=1}^{2n_z+1} w_i (\mathcal{Z}_i - \tilde{\mu}_t)(\mathcal{Z}_i - \bar{z})^T$ ▷ Compute cross-covariance D
  $G_t \leftarrow D P^{-1}$ ▷ Compute smoother gain G_t
  $\hat{\mu}_t \leftarrow \tilde{\mu}_t + G_t \left(\hat{\mu}_{t+1} - \bar{z}\right)$ ▷ Compute smoothed mean $\hat{\mu}_t$
  $\hat{V}_t \leftarrow \tilde{V}_t + \left(G_t \hat{V}_{t+1} - D\right) {G_t}^T$ ▷ Compute smoothed covariance $\hat{V}_t$
  return $\hat{\mu}_t, \hat{V}_t, G_t$

To obtain values of the smoothed means $\hat{\mu} = \{\hat{\mu}_0, ..., \hat{\mu}_T\}$ and covariances $\hat{V} = \{\hat{V}_0, ..., \hat{V}_T\}$ for all time points one needs to run the forward filtering path and then iterate backwards through the entire behavioral sequence:

Algorithm 9

function $\mathrm{f}_{\mathrm{uks}}(\mu_0, V_0, V_z, V_x)$
  $\tilde{\mu}, \tilde{V} \leftarrow \mathrm{f}_{ukf}\left(\mu_0, V_0, V_z, V_x\right)$
  $\hat{\mu}_T \leftarrow \tilde{\mu}_T$
  $\hat{V}_T \leftarrow \tilde{V}_T$
  for $t \in \{T-1, ..., 0\}$ **do**
    $\hat{\mu}_t, \hat{V}_t, G_t \leftarrow \mathrm{f}_{uks_0}\left(\tilde{\mu}_t, \tilde{V}_t, \hat{\mu}_{t+1}, \hat{V}_{t+1}, V_z\right)$
  return $\hat{\mu}, \hat{V}, G$

Here, the set of all smoother gains $G = \{G_0, ..., G_{T-1}\}$ is needed for performing the M-step later on.

4.4.3. Enforcing anatomical constraints

The plain formulation of the unscented RTS smoother does not allow constraining the state variables. However, in order to enforce joint angle limits we need to ensure that Rodrigues vectors encoding bone rotations stay within specified limits. Therefore, we introduce a mapping function $\mathrm{f}_{\mathrm{z}^* \to \mathrm{z}}$, which allows for mapping a redefined state variable $z_t^* \in \mathbb{R}^{n_z}$ onto the original one $z_t \in \mathbb{R}^{n_z}$, while enforcing that entries of z_t corresponding to bone rotations stay within their respective lower and upper bounds. The respective mapping function $\mathrm{f}_{\mathrm{z}^* \to \mathrm{z}}$ is given by Algorithm 10.

Algorithm 10

function $\mathrm{f}_{\mathrm{z}^* \to \mathrm{z}}(z_t^*)$
  $t^* \leftarrow \left(z_{t\,1}^*, z_{t\,2}^*, z_{t\,3}^*\right)^T$ ▷ Obtain normalized global translation t^*
  $r_1^* \leftarrow \left(z_{t\,4}^*, z_{t\,5}^*, z_{t\,6}^*\right)^T$ ▷ Obtain normalized global rotation r_1^*
  for $i \in \{2, .., n_{\mathrm{bone}}\}$ **do**
    $r_i^{**} \leftarrow \left(z_{t\,3i+1}^*, z_{t\,3i+2}^*, z_{t\,3i+3}^*\right)^T$ ▷ Obtain redefined bone rotation r_i^{**}
    for $j \in \{1, .., 3\}$ **do**
      $n \leftarrow \mathrm{erf}\left(\frac{\sqrt{\pi}}{2} r_{ij}^{**}\right)$ ▷ Map $r_{ij}^{**} \in (-\inf, \inf)$ to $n \in (-1, 1)$
      $r_{ij} \leftarrow b_{0ij} + \frac{1}{2}\left(b_{1ij} - b_{0ij}\right)(1 + n)$ ▷ Compute $r_{ij} \in \left(b_{0ij}, b_{1ij}\right)$
    $r_i^* \leftarrow \frac{r_i}{c_r}$ ▷ Obtain normalized bone rotation r_i^*
  $z_t \leftarrow \left(t^*, r_1^*, r_2^*, ..., r_{n_{\mathrm{bone}}}^*\right)^T$ ▷ Obtain z_t via concatenation
  return z_t

Here, b_{0ij} and b_{1ij} denote the lower and upper bound corresponding to entry j of the Rodrigues vector r_i, which encodes the rotation of bone i, and erf is a sigmoidal function, i.e. the error function given by:

$$\mathrm{erf}\,(y) = \frac{2}{\sqrt{\pi}} \int_0^y \exp\left(-t^2\right) dt \tag{48}$$

for $y \in \mathbb{R}$. In order to enforce joint angle limits we just replace the original transmission and emission equation in our state space model given by equations 13 and 14 with:

$$z_t^* = z_{t-1}^* + \epsilon_z \tag{49}$$

$$x_t = \mathrm{g}\left(\mathrm{f}_{\mathrm{z}^* \to \mathrm{z}}\left(z_t^*\right)\right) + \epsilon_x. \tag{50}$$

In the following we always refer to the state space model given by equations 49 and 50 and therefore also to the redefined state variables z^* but we drop * in the notation.

4.5. Maximization step

In the M-step we find a new set of model parameters Θ_{k+1} by maximizing the ELBO $\mathcal{L}$, given the smoothed means $\hat{\mu}$ and covariances $\hat{V}$ as well as the smoother gains G, which we obtained in the E-step using a current estimate of the model parameters Θ_k.

4.5.1. Obtaining new model parameters by maximizing the evidence lower bound

We can take advantage of the specific structure of the state space model when maximizing the ELBO $\mathcal{L}$ [8]. In the state space model the state variables fulfill the Markov property, i.e. each state variable z_t only depends on the previous one z_{t-1}. Based on this we can compute the model's joint distribution:

$$\mathrm{p}\left(x, z\right) = \mathrm{p}\left(z_0\right) \prod_{t=1}^{T} \mathrm{p}\left(z_t | z_{t-1}\right) \mathrm{p}\left(x_t | z_t\right). \tag{51}$$

When we now take the logarithm of the joint distribution and acknowledge that the model parameters Θ are also required for computing the joint distribution we obtain:

$$\ln \mathrm{p}\left(x, z | \Theta\right) = \ln \mathrm{p}\left(z_0 | \mu_0, V_0\right) + \sum_{t=1}^{T} \ln \mathrm{p}\left(z_t | z_{t-1}, V_z\right) + \sum_{t=1}^{T} \ln \mathrm{p}\left(x_t | z_t, V_x\right). \tag{52}$$

However, to maximize $\mathcal{Q}\left(\Theta, \Theta_k\right)$ we actually need to consider the expectation value of $\ln \mathrm{p}\left(x, z | \Theta\right)$:

$$\mathcal{Q}\left(\Theta, \Theta_k\right) = \mathbb{E}\left[\ln \mathrm{p}\left(x, z | \Theta\right)\right] \tag{53}$$

$$= \mathbb{E}\left[\ln \mathrm{p}\left(z_0 | \mu_0, V_0\right)\right] + \sum_{t=1}^{T} \mathbb{E}\left[\ln \mathrm{p}\left(z_t | z_{t-1}, V_z\right)\right] + \sum_{t=1}^{T} \mathbb{E}\left[\ln \mathrm{p}\left(x_t | z_t, V_x\right)\right] \tag{54}$$

$$= I_0 + I_z + I_x \tag{55}$$

with $I_0 = \mathbb{E}\left[\ln \mathrm{p}\left(z_0 | \mu_0, V_0\right)\right]$, $I_z = \sum_{t=1}^{T} \mathbb{E}\left[\ln \mathrm{p}\left(z_t | z_{t-1}, V_z\right)\right]$ and $I_x = \sum_{t=1}^{T} \mathbb{E}\left[\ln \mathrm{p}\left(x_t | z_t, V_x\right)\right]$. If we now acknowledge that all random variables in our state space model are normally distributed, i.e. $z_t \sim \mathcal{N}\left(\hat{\mu}_t, \hat{V}_t\right)$, it becomes clear that computing $\mathcal{Q}\left(\Theta, \Theta_k\right)$ only involves evaluating the expectation values of log-transformed normal distributions (see Appendix A). Consequently, we can obtain simplified terms for the individual components I_0, I_z and I_x of $\mathcal{Q}\left(\Theta, \Theta_k\right)$ using the smoothed means $\hat{\mu}$ and covariances $\hat{V}$ as well as the smoother gains G. For I_0 we get:

$$I_0 = -\frac{1}{2} \ln \det\left(2\pi V_0\right) - \frac{1}{2} \mathrm{tr}\left(V_0^{-1} \mathbb{E}\left[\left(z_0 - \mu_0\right)\left(z_0 - \mu_0\right)^T\right]\right) \tag{56}$$

$$= -\frac{1}{2} \ln \det\left(2\pi V_0\right) - \frac{1}{2} \mathrm{tr}\left(V_0^{-1}\left(\hat{V}_0 + \left(\hat{\mu}_0 - \mu_0\right)\left(\hat{\mu}_0 - \mu_0\right)^T\right)\right). \tag{57}$$

To obtain a simplified expression for I_z we need to form pairwise sigma points $\mathcal{P}_t$, as there are always two random variables involved simultaneously, $z_t \sim \mathcal{N}\left(\hat{\mu}_t, \hat{V}_t\right)$ and $z_{t-1} \sim \mathcal{N}\left(\hat{\mu}_{t-1}, \hat{V}_{t-1}\right)$, when evaluating the expectation values of the underlying log-transformed normal distributions in I_z. For each of the T transition steps we generate the pairwise mean vector $\breve{\mu}_t \in \mathbb{R}^{2n_z}$:

$$\breve{\mu}_t = \begin{pmatrix} \hat{\mu}_t \\ \hat{\mu}_{t-1} \end{pmatrix} \tag{58}$$

as well as the pairwise covariance matrix $\breve{V}_t \in \mathbb{R}^{2n_z \times 2n_z}$:

$$\breve{V}_t = \begin{pmatrix} \hat{V}_t & \hat{V}_t {G_{t-1}}^T \\ G_{t-1}\hat{V}_t & \hat{V}_{t-1} \end{pmatrix} \tag{59}$$

and calculate the pairwise sigma points $\mathcal{P}_t$ as follows:

$$\mathcal{P}_t = \begin{pmatrix} \mathcal{B}_t \\ \mathcal{A}_t \end{pmatrix} = \mathrm{f}_{\mathrm{ut}}\left(\breve{\mu}_t, \breve{V}_t\right) \tag{60}$$

where concatenating the incomplete pairwise sigma points $\mathcal{B}_t \in \mathbb{R}^{4n_z+1 \times n_z}$ and $\mathcal{A}_t \in \mathbb{R}^{4n_z+1 \times n_z}$ gives $\mathcal{P}_t \in \mathbb{R}^{4n_z+1 \times 2n_z}$. Consequently, the weights $\breve{w}$ associated with the pairwise sigma points $\mathcal{P}_t$ are then given in accordance with the concepts discussed in Section 4.3:

$$\breve{w}_1 = 0 \tag{61}$$

$$\breve{w}_i = \frac{1}{4n_z} \forall i \in \{2, ..., 4n_z + 1\} \tag{62}$$

A simplified term for I_z is then given by:

$$I_z = -\frac{T}{2} \ln\det\left(2\pi V_z\right) - \frac{1}{2}\sum_{t=1}^{T} \mathrm{tr}\left({V_z}^{-1}\mathbb{E}\left[\left(z_t - z_{t-1}\right)\left(z_t - z_{t-1}\right)^T\right]\right) \tag{63}$$

$$= -\frac{T}{2} \ln\det\left(2\pi V_z\right) - \frac{T}{2} \mathrm{tr}\left({V_z}^{-1}\left(\frac{1}{T}\sum_{t=1}^{T}\mathbb{E}\left[\left(z_t - z_{t-1}\right)\left(z_t - z_{t-1}\right)^T\right]\right)\right) \tag{64}$$

$$\approx -\frac{T}{2} \ln\det\left(2\pi V_z\right) - \frac{T}{2}\sum_{t=1}^{T} \mathrm{tr}\left({V_z}^{-1}\left(\frac{1}{T}\sum_{i=1}^{4n_z+1}\breve{w}_i\left(\mathcal{B}_{ti} - \mathcal{A}_{ti}\right)\left(\mathcal{B}_{ti} - \mathcal{A}_{ti}\right)^T\right)\right). \tag{65}$$

To evaluate the expectation value in I_x it is sufficient to just use the normal sigma points $\mathcal{Z}_t = \mathrm{f}_{\mathrm{ut}}\left(\hat{\mu}_t, \hat{V}_t\right)$ and propagate them through our emission function g:

$$I_x = -\frac{T}{2} \ln\det\left(2\pi V_x\right) - \frac{1}{2}\sum_{t=1}^{T} \mathrm{tr}\left({V_x}^{-1}\mathbb{E}\left[\left(x_t - \mathrm{g}\left(z_t\right)\right)\left(x_t - \mathrm{g}\left(z_t\right)\right)^T\right]\right) \tag{66}$$

$$= -\frac{T}{2} \ln\det\left(2\pi V_x\right) - \frac{T}{2} \mathrm{tr}\left({V_x}^{-1}\left(\frac{1}{T}\sum_{t=1}^{T}\mathbb{E}\left[\left(x_t - \mathrm{g}\left(z_t\right)\right)\left(x_t - \mathrm{g}\left(z_t\right)\right)^T\right]\right)\right) \tag{67}$$

$$\approx -\frac{T}{2} \ln\det\left(2\pi V_x\right) - \frac{T}{2}\sum_{t=1}^{T} \mathrm{tr}\left({V_x}^{-1}\left(\frac{1}{T}\sum_{i=1}^{2n_z+1} w_i\left(x_t - \mathrm{g}\left(\mathcal{Z}_{ti}\right)\right)\left(x_t - \mathrm{g}\left(\mathcal{Z}_{ti}\right)\right)^T\right)\right). \tag{68}$$

To finally obtain new model parameters $\Theta_{k+1} = \{\mu_{0,k+1}, V_{0,k+1}, V_{z,k+1}, V_{x,k+1}\}$ we still need to differentiate $\mathcal{Q}\left(\Theta, \Theta_k\right)$ with respect to μ_0, V_0, V_z and V_x, set the resulting derivatives to zero and solve them

for μ_0, V_0, V_z and V_x respectively. The required derivatives have the following form (see Appendix B):

$$\frac{d}{d\mu_0}\mathcal{Q}(\Theta,\Theta_k) = \frac{d}{d\mu_0}I_0 \tag{69}$$

$$= V_0^{-1}(\hat{\mu}_0 - \mu_0) \tag{70}$$

$$\frac{d}{dV_0}\mathcal{Q}(\Theta,\Theta_k) = \frac{d}{dV_0}I_0 \tag{71}$$

$$= -\frac{1}{2}V_0^{-1} + \frac{1}{2}V_0^{-1}\left(\hat{V}_0 + (\hat{\mu}_0 - \mu_0)(\hat{\mu}_0 - \mu_0)^T\right)V_0^{-1} \tag{72}$$

$$\frac{d}{dV_z}\mathcal{Q}(\Theta,\Theta_k) = \frac{d}{dV_z}I_z \tag{73}$$

$$= -\frac{T}{2}V_z^{-1} + \frac{T}{2}\sum_{t=1}^{T}V_z^{-1}\left(\frac{1}{T}\sum_{i=1}^{4n_z+1}\breve{w}_i(\mathcal{B}_{ti} - \mathcal{A}_{ti})(\mathcal{B}_{ti} - \mathcal{A}_{ti})^T\right)V_z^{-1} \tag{74}$$

$$\frac{d}{dV_x}\mathcal{Q}(\Theta,\Theta_k) = \frac{d}{dV_x}I_x \tag{75}$$

$$= -\frac{T}{2}V_x^{-1} + \frac{T}{2}\sum_{t=1}^{T}V_x^{-1}\left(\frac{1}{T}\sum_{i=1}^{2n_z+1}w_i(x_t - \mathrm{g}(\mathcal{Z}_{ti}))(x_t - \mathrm{g}(\mathcal{Z}_{ti}))^T\right)V_x^{-1}. \tag{76}$$

Setting these derivatives to zero and solving for μ_0, V_0, V_z and V_x yields the following:

$$\mu_0 = \hat{\mu}_0 \tag{77}$$

$$V_0 = \hat{V}_0 + (\hat{\mu}_0 - \mu_0)(\hat{\mu}_0 - \mu_0)^T \tag{78}$$

$$V_z = \frac{1}{T}\sum_{t=1}^{T}\sum_{i=1}^{4n_z+1}\breve{w}_i(\mathcal{B}_{ti} - \mathcal{A}_{ti})(\mathcal{B}_{ti} - \mathcal{A}_{ti})^T \tag{79}$$

$$V_x = \frac{1}{T}\sum_{t=1}^{T}\sum_{i=1}^{2n_z+1}w_i(x_t - \mathrm{g}(\mathcal{Z}_{ti}))(x_t - \mathrm{g}(\mathcal{Z}_{ti}))^T. \tag{80}$$

The resulting values for $\mu_{0,k+1}$, $V_{z,k+1}$ and $V_{x,k+1}$ are then given by equations 77, 79 and 80. To obtain $V_{0,k+1}$ we need to substitute $\mu_{0,k+1}$ into equation 78, giving $V_{0,k+1} = \hat{V}_0$. Lastly, we still need to adjust the solution for $V_{x,k+1}$ to also account for missing measurements. Besides that, we note that it is sufficient to only compute the diagonal entries of $V_{x,k+1}$, since we enforce the covariance matrix of the measurement noise V_x to be a diagonal matrix. Thus, the final solution for a diagonal entry $j \in \{1, ..., n_x\}$ of $V_{x,k+1}$ is given by:

$$\mathrm{diag}(V_{x,k+1})_j = \frac{1}{T_j}\sum_{t=1}^{T}\delta_{tj}\sum_{i=1}^{2n_z+1}w_i\Big(x_{tj} - \mathrm{g}(\mathcal{Z}_{ti})_j\Big)^2 \tag{81}$$

where the function diag gives the diagonal entries of the input matrix, δ_{tj} indicates if at time point t the entry j of x_t is associated with a missing measurement, i.e. $\delta_{tj} = 0$, or not, i.e. $\delta_{tj} = 1$, and T_j is the total number of successful measurements for entry j in the entire behavioral sequence, i.e. $T_j = \sum_{t=1}^{T}\delta_{tj}$.

4.6. Convergence of the expectation-maximization algorithm

We calculate the changes in the model parameters Θ in each iteration k of the EM algorithm to check for convergence [1]. Particularly, we are computing the vectors $\Delta\mu_0 \in \mathbb{R}^{n_z}$, $\Delta\,\mathrm{diag}(V_0) \in \mathbb{R}^{n_z}$, $\Delta\,\mathrm{diag}(V_z) \in \mathbb{R}^{n_z}$ and $\Delta\,\mathrm{diag}(V_x) \in \mathbb{R}^{n_x}$, which contain the relative changes of μ_0, V_0, V_z and V_x at

iteration k:

$$\Delta\mu_{0i} = \text{abs}\left(\frac{\mu_{0,k_i} - \mu_{0,k-1_i}}{\mu_{0,k-1_i}}\right) \ \forall\, i \in \{1, ..., n_z\} \tag{82}$$

$$\Delta\,\text{diag}\,(V_0)_i = \text{abs}\left(\frac{V_{0,k_{ii}} - V_{0,k-1_{ii}}}{V_{0,k-1_{ii}}}\right) \ \forall\, i \in \{1, ..., n_z\} \tag{83}$$

$$\Delta\,\text{diag}\,(V_z)_i = \text{abs}\left(\frac{V_{z,k_{ii}} - V_{z,k-1_{ii}}}{V_{z,k-1_{ii}}}\right) \ \forall\, i \in \{1, ..., n_z\} \tag{84}$$

$$\Delta\,\text{diag}\,(V_x)_i = \text{abs}\left(\frac{V_{x,k_{ii}} - V_{x,k-1_{ii}}}{V_{x,k-1_{ii}}}\right) \ \forall\, i \in \{1, ..., n_x\} \tag{85}$$

where abs is a function returning the absolute value of its input argument and $\mu_{0,k}$, $V_{0,k}$, $V_{z,k}$ and $V_{x,k}$ are the model parameters at iteration k whereas $\mu_{0,k-1}$, $V_{0,k-1}$, $V_{z,k-1}$ and $V_{x,k-1}$ are those at iteration $k-1$. We only focus on the diagonal entries of the covariances V_0 and V_z since a fraction of their off-diagonal entries is expected to be zero. Using these relative changes we construct a vector $\Delta v \in \mathbb{R}^{3n_z+n_x}$ containing all relative changes via concatenation:

$$\Delta v = \left(\Delta\mu_0, \Delta\,\text{diag}\,(V_0)\,, \Delta\,\text{diag}\,(V_z)\,, \Delta\,\text{diag}\,(V_x)\right)^T \tag{86}$$

and assume convergence is reached when the mean $\Delta\bar{v}$ of Δv falls below a threshold ϵ_{tol}:

$$\Delta\bar{v} = \frac{1}{3n_z + n_x} \sum_{i=1}^{3n_z+n_x} \Delta v_i < \epsilon_{\text{tol}} \tag{87}$$

where we set $\epsilon_{\text{tol}} = 0.05$.

4.7. Implementation of the expectation-maximization algorithm

We initialize the mean of the state variables μ_0 by minimizing the objective function given by equation 9 but keep the bone lengths l and the surface marker positions v constant and set $n_{\text{time}} = 1$, i.e. we only include a single time point in the optimization, which is identical to the first time point of a respective behavioral sequence. The covariances V_0, V_x and V_z are initialized as matrices whose diagonal elements all equal 0.001 and off-diagonal entries are set to zero. To learn new model parameters μ_0, V_0, V_x and V_z we run the EM algorithm, given by Algorithm 11, with the stated initial values, i.e. $V_{0,0}$, $V_{z,0}$ and $V_{x,0}$, using measurements x obtained from the behavioral sequence. Finally, once the EM algorithm converged, we use the unscented RTS smoother with the resulting learned model parameters to reconstruct poses of the behavioral sequence.

Algorithm 11

function $\text{f}_{\text{EM}}(\mu_0, V_0, V_z, V_x)$
  $k \leftarrow 0$ ▷ Initialize iteration number k
  $\Delta\bar{v} \leftarrow$ inf
  while $\Delta\bar{v} \geq \epsilon_{\text{tol}}$ **do**
    $\hat{\mu}, \hat{V}, G \leftarrow \text{f}_{\text{uks}}\left(\mu_{0,k}, V_{0,k}, V_{z,k}, V_{x,k}\right)$ ▷ Perform E-step
    $\mu_{0,k+1}, V_{0,k+1}, V_{z,k+1}, V_{x,k+1} \leftarrow \text{f}_{\text{M}}\left(\hat{\mu}, \hat{V}, G\right)$ ▷ Perform M-step
    $k \leftarrow k + 1$ ▷ Increase iteration number k
    $\Delta\bar{v} \leftarrow \text{f}_{\text{tol}}\left(\mu_{0,k-1}, V_{0,k-1}, V_{z,k-1}, V_{x,k-1}, \mu_{0,k}, V_{0,k}, V_{z,k}, V_{x,k}\right)$ ▷ Compute change in Θ
  return $\mu_{0,k}, V_{0,k}, V_{z,k}, V_{x,k}$

Here, in accordance to the concepts stated in Section 4.5 and 4.6, function f_{M}, given by Algorithm 12, performs the M-step and function f_{tol}, given by Algorithm 13, computes the mean $\Delta\bar{v}$ of the relative changes of the model parameters Θ.

Algorithm 12

function $\mathrm{f_M}(\hat{\mu}, \hat{V}, G)$

  for $t \in \{1, ..., T\}$ **do**

    $\begin{pmatrix} \mathcal{B}_t \\ \mathcal{A}_t \end{pmatrix} \leftarrow \mathrm{f_{ut}}\left(\begin{pmatrix} \hat{\mu}_t \\ \hat{\mu}_{t-1} \end{pmatrix}, \begin{pmatrix} \hat{V}_t & \hat{V}_t {G_{t-1}}^T \\ G_{t-1}\hat{V}_t & \hat{V}_{t-1} \end{pmatrix}\right)$

    $\mathcal{Z}_t \leftarrow \mathrm{f_{ut}}\left(\hat{\mu}_t, \hat{V}_t\right)$

  $\mu_{0,k+1} \leftarrow \hat{\mu}_0$

  $V_{0,k+1} \leftarrow \hat{V}_0$

  $V_{z,k+1} \leftarrow \frac{1}{T} \sum_{t=1}^{T} \sum_{i=1}^{4n_z+1} \breve{w}_i \left(\mathcal{B}_{ti} - \mathcal{A}_{ti}\right)\left(\mathcal{B}_{ti} - \mathcal{A}_{ti}\right)^T$

  for $j \in \{1, ..., n_x\}$ **do**

    ${V_{x,k+1}}_{jj} \leftarrow \frac{1}{T_j} \sum_{t=1}^{T} \delta_{tj} \sum_{i=1}^{2n_z+1} w_i \left(x_{tj} - \mathrm{g}\left(\mathcal{Z}_{ti}\right)_j\right)^2$

  return $\mu_{0,k+1}, V_{0,k+1}, V_{z,k+1}, V_{x,k+1}$

Algorithm 13

function $\mathrm{f_{tol}}(\mu_{0,k-1}, V_{0,k-1}, V_{z,k-1}, V_{x,k-1}, \mu_{0,k}, V_{0,k}, V_{z,k}, V_{x,k})$

  for $i \in \{1, ..., n_z\}$ **do**

    $\Delta\mu_{0i} \leftarrow \mathrm{abs}\left(\frac{\mu_{0,k_i} - \mu_{0,k-1_i}}{\mu_{0,k-1_i}}\right)$

    $\Delta\,\mathrm{diag}\left(V_0\right)_i \leftarrow \mathrm{abs}\left(\frac{V_{0,k_{ii}} - V_{0,k-1_{ii}}}{V_{0,k-1_{ii}}}\right)$

    $\Delta\,\mathrm{diag}\left(V_z\right)_i \leftarrow \mathrm{abs}\left(\frac{V_{z,k_{ii}} - V_{z,k-1_{ii}}}{V_{z,k-1_{ii}}}\right)$

  for $i \in \{1, ..., n_x\}$ **do**

    $\Delta\,\mathrm{diag}\left(V_x\right)_i \leftarrow \mathrm{abs}\left(\frac{V_{x,k_{ii}} - V_{x,k-1_{ii}}}{V_{x,k-1_{ii}}}\right)$

  $\Delta v \leftarrow \left(\Delta\mu_0, \Delta\,\mathrm{diag}\left(V_0\right), \Delta\,\mathrm{diag}\left(V_z\right), \Delta\,\mathrm{diag}\left(V_x\right)\right)^T$

  $\Delta\bar{v} \leftarrow \frac{1}{3n_z+n_x} \sum_{i=1}^{3n_z+n_x} \Delta v_i$

  return $\Delta\bar{v}$

5. Training deep neural networks to detect 2D locations of surface markers

To automatically detect 2D locations of surface makers we used DeepLabCut [9]. For each rat or mouse in each dataset an individual neural network was trained on manually labeled images obtained from four different cameras. For training, each image was cropped to 600x600 px^2 around a centerpoint determined by background subtraction: For each training image, a background-subtracted image was generated by subtracting the image acquired 200 ms prior to the frame of interest for the FTIR and gap-crossing datasets and 125 ms prior for the gait dataset. Subsequently, approximate 2D locations of the recorded animal on the background-subtracted images were obtained by calculating the median indices of pixels above a threshold-value of 5 times the standard deviation of each pixel, where the standard deviations were calculated from the first 100 images of each recorded video, which were acquired with the arena or track empty and free of any moving objects. These 2D locations were then used as a center-point to crop the original images to 600x600 px^2. To minimize the influence of visible movements of the experimenters on this center-point detection in the recorded FTIR data set, pixel values of pixels, which did not show the FTIR plate, were set to zero for the recordings of animals #3 to #6. Resulting images that did not contain any manually annotated 2D positions of surface markers due to the preprocessing steps not leading to correct cropping, were not used during training.

For the rat FTIR datasets the networks were trained on 4068 images for animal #1, 3980 images for animal #2, 752 images for animal #3, 1100 images for animal #4, 992 images for animal

#5 and 1128 images for animal #6. For the rat gait and gap-crossing datasets 2404 and 3608 images were used respectively for each analyzed animal (animal #1 and #2). For the rat data with additional IMUs 904 and 2520 images were used for animal # 7 and #8 respectively. For the experiments with mice, 808 and 1364 images were used for mouse #1 and #2 respectively. We used DeepLabCut's default settings, with the only two exceptions being that we changed the network architectures to ResNet-152 and enabled mirroring of images for which we paired surface markers with a left/right correspondence78. For the rat gait, gap-crossing and FTIR datasets, training was conducted via DeepLabCut 2.1.6.4 and (for the rat IMU and mouse datasets) 2.1.10.4 (https://github.com/DeepLabCut/DeepLabCut/releases/). Subsequently we used DLC to obtain 2D locations of surface markers in all frames of behavioral sequences, to 0.9 and treated detected marker positions below this value as missing measurements.discarding any detections with a confidence value below 0.9.

6. Box constraints for surface marker positions based on body symmetry

Box constraints for central and left-sided surface maker locations were defined in the coordinate system of the associated edges and set as follows

marker	attached joint	x	y	z
head #1	spine #5	[0,0]	[0,inf)	(-inf,inf)
head #2	spine #5	[0,0]	[0,inf)	(-inf,inf)
head #3	head (leaf)	[0,0]	[0,0)	[0,0]
spine #1	spine #2	[0,0]	[0,inf)	(-inf,inf)
spine #2	spine #2	[0,0]	[0,inf)	(-inf,inf)
spine #3	spine #3	[0,0]	[0,inf)	(-inf,inf)
spine #4	spine #3	[0,0]	[0,inf)	(-inf,inf)
spine #5	spine #4	[0,0]	[0,inf)	(-inf,inf)
spine #6	spine #5	[0,0]	[0,inf)	[0,0]
tail #1	tail #1 (leaf)	[0,0]	[0,0]	[0,0]
tail #2	tail #2	[0,0]	[0,inf)	(-inf,inf)
tail #3	tail #3	[0,0]	[0,inf)	(-inf,inf)
tail #4	tail #4	[0,0]	[0,inf)	(-inf,inf)
tail #5	tail #5	[0,0]	[0,inf)	(-inf,inf)
tail #6	spine #1	[0,0]	[0,inf)	(-inf,inf)
shoulder	shoulder	[0,0]	[0,inf)	[0,inf)
elbow	elbow	(-inf,0]	[0,0]	[0,0]
wrist	wrist	[0,0]	(-inf,0]	[0,0]
finger #1	finger (leaf)	(-inf,inf)	[0,0]	(-inf,inf)
finger #2	finger (leaf)	[0,0]	[0,0]	[0,0]
finger #3	finger (leaf)	(-inf,inf)	[0,0]	(-inf,inf)
side	spine #3	(-inf,0]	(-inf,inf)	(-inf,inf)
hip	hip	[0,0]	[0,inf)	[0,inf)
knee	knee	(-inf,0]	[0,0]	[0,0]
ankle	ankle	(-inf,0]	[0,0]	[0,0]
metatarsophalangeal	metatarsophalangeal	[0,0]	(-inf,0]	[0,0]
toe #1	toe (leaf)	(-inf,inf)	[0,0]	(-inf,inf)
toe #2	toe (leaf)	[0,0]	[0,0]	[0,0]
toe #3	toe (leaf)	(-inf,inf)	[0,0]	(-inf,inf)

Appendix

A. Evaluating expected values of log-transformed normal distributions

Given a d-dimensional normal distribution $\mathrm{p}_{\mathrm{norm}}$ with mean $\mu_y \in \mathbb{R}^d$ and covariance $V_y \in \mathbb{R}^{d \times d}$, evaluating it for a normally distributed random variable $y \sim \mathcal{N}(m, \Sigma)$ takes the following form:

$$\mathrm{p}_{\mathrm{norm}}(y|\mu_y, V_y) = (2\pi)^{-\frac{d}{2}} \det(V_y)^{-\frac{1}{2}} \exp\left(-\frac{1}{2}(y-\mu_y)^T {V_y}^{-1}(y-\mu_y)\right) \tag{88}$$

where $\det(V_y) \in \mathbb{R}$ denotes the determinant of matrix V_y. Applying a logarithmic transformation yields:

$$\ln \mathrm{p}_{\mathrm{norm}}(y|\mu_y, V_y) = -\frac{1}{2}\ln\det(2\pi V_y) - \frac{1}{2}\mathrm{tr}\left({V_y}^{-1}(y-\mu_y)(y-\mu_y)^T\right) \tag{89}$$

where $\mathrm{tr}\left(V_y\right) \in \mathbb{R}$ denotes the trace of matrix V_y. Noticing that $\mathbb{E}\left[yy^T\right] = \Sigma + mm^T$ [8], we can take the expected value of equation 89 with respect to y and obtain:

$$\mathbb{E}\left[\ln \mathrm{p}_{\mathrm{norm}}\left(y|\mu_y, V_y\right)\right] = -\frac{1}{2}\ln\det\left(2\pi V_y\right) - \frac{1}{2}\mathrm{tr}\left({V_y}^{-1}\mathbb{E}\left[\left(y-\mu_y\right)\left(y-\mu_y\right)^T\right]\right) \tag{90}$$

$$= -\frac{1}{2}\ln\det\left(2\pi V_y\right) - \frac{1}{2}\mathrm{tr}\left({V_y}^{-1}\left(\Sigma + \left(m-\mu_y\right)\left(m-\mu_y\right)^T\right)\right) \tag{91}$$

B. Derivatives

Given a d-dimensional vector $v \in \mathbb{R}^d$, two symmetric matrices $M \in \mathbb{R}^{d\times d}$ and $C \in \mathbb{R}^{d\times d}$ as well as a scalar $c \in \mathbb{R}$, we can obtain the following derivatives:

$$\frac{d}{dv}\mathrm{tr}\left(Cvv^T\right) = Cv + C^T v = 2Cv \tag{92}$$

$$\frac{d}{dM}\ln\det\left(cM\right) = M^{-1} \tag{93}$$

$$\frac{d}{dM}\mathrm{tr}\left(M^{-1}C\right) = -\left(M^T\right)^{-1}C^T\left(M^T\right)^{-1} = -M^{-1}CM^{-1}. \tag{94}$$

References

[1] Christopher M. Bishop. *Pattern Recognition and Machine Learning*. Springer, 2006.

[2] Mary Ann Branch, Thomas F. Coleman, and Yuying Li. A subspace, interior, and conjugate gradient method for large-scale bound-constrained minimization problems. *SIAM Journal on Scientific Computing*, 21(1):1–23, 1999.

[3] Richard H. Byrd, Peihuang Lu, Jorge Nocedal, and Ciyou Zhu. A limited memory algorithm for bound constrained optimization. *SIAM Journal of Scientific Computing*, 16:1190–1208, September 1995.

[4] J. Chen, S. Nie, and Q. Ji. Data-free prior model for upper body pose estimation and tracking. *IEEE Transactions on Image Processing*, 22(12):4627–4639, 2013.

[5] A. P. Dempster, N. M. Laird, and D. B. Rubin. Maximum likelihood from incomplete data via the em algorithm. *Journal of the Royal Statistical Society. Series B (Methodological)*, 39(1):1–38, 1977.

[6] Intel. *The OpenCV Reference Manual*, 4.5.2 edition, 2021.

[7] S. J. Julier, J. K. Uhlmann, and H. F. Durrant-Whyte. A new approach for filtering nonlinear systems. In *Proceedings of 1995 American Control Conference - ACC'95*, volume 3, pages 1628–1632 vol.3, 1995.

[8] Juho Kokkala, Arno Solin, and Simo Särkkä. Sigma-point filtering and smoothing based parameter estimation in nonlinear dynamic systems, 2015.

[9] Alexander Mathis, Pranav Mamidanna, Kevin M. Cury, Taiga Abe, Venkatesh N. Murthy, Mackenzie W. Mathis, and Matthias Bethge. Deeplabcut: markerless pose estimation of user-defined body parts with deep learning. *Nature Neuroscience*, 2018.

[10] Kevin P. Murphy. *Machine Learning: A Probabilistic Perspective*. The MIT Press, 2012.

[11] Gerard Pons-Moll and Bodo Rosenhahn. Ball joints for marker-less human motion capture. In *IEEE Workshop on Applications of Computer Vision (WACV)*, December 2009.

[12] Robert H. Shumway and David S. Stoffer. *Time Series Analysis and Its Applications*. Springer, 4th edition, 2017.

[13] Simo Särkkä. *Bayesian Filtering and Smoothing*. Institute of Mathematical Statistics Textbooks. Cambridge University Press, 2013.

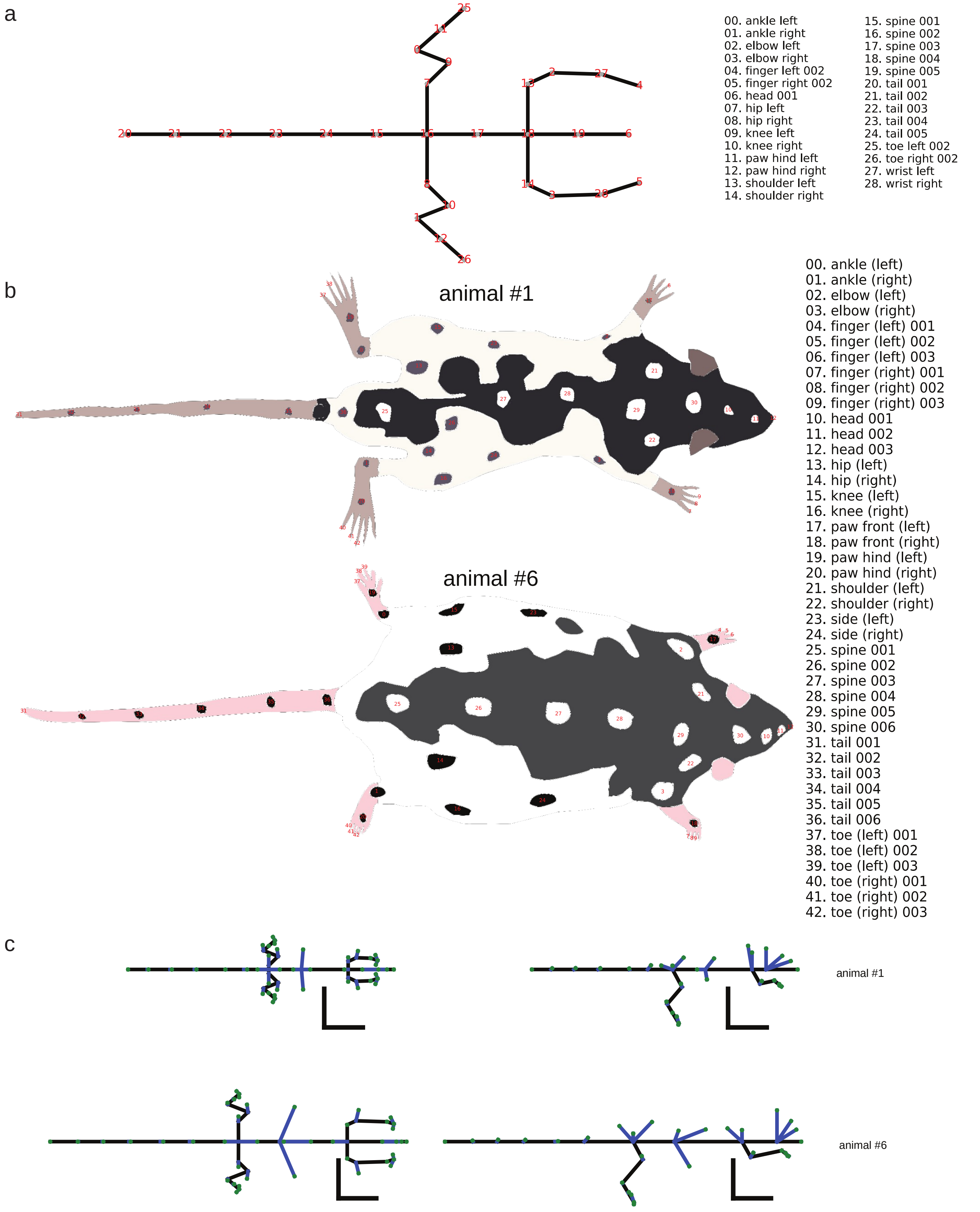

Supplementary Fig. 1 | a, Projection of the generalized skeleton model in the xy-plane with index and name for each individual joint. For this figure, all bone lengths are equal and all bone rotations have been set to the mean of their upper and lower bounds. **b**, Schematic images of the two of the six labeled animals with index and name for each individual surface marker. **c,** Projections of the generalized and learned skeleton models two of the six rats as viewed from the top (xy-view, left column) and from the side (xz-view, right column). All bone rotations were set to the mean of their upper and lower bounds. Green dots indicate the learned positions of surface markers and blue lines join paired joints and surface markers. All scale bars 5 cm.

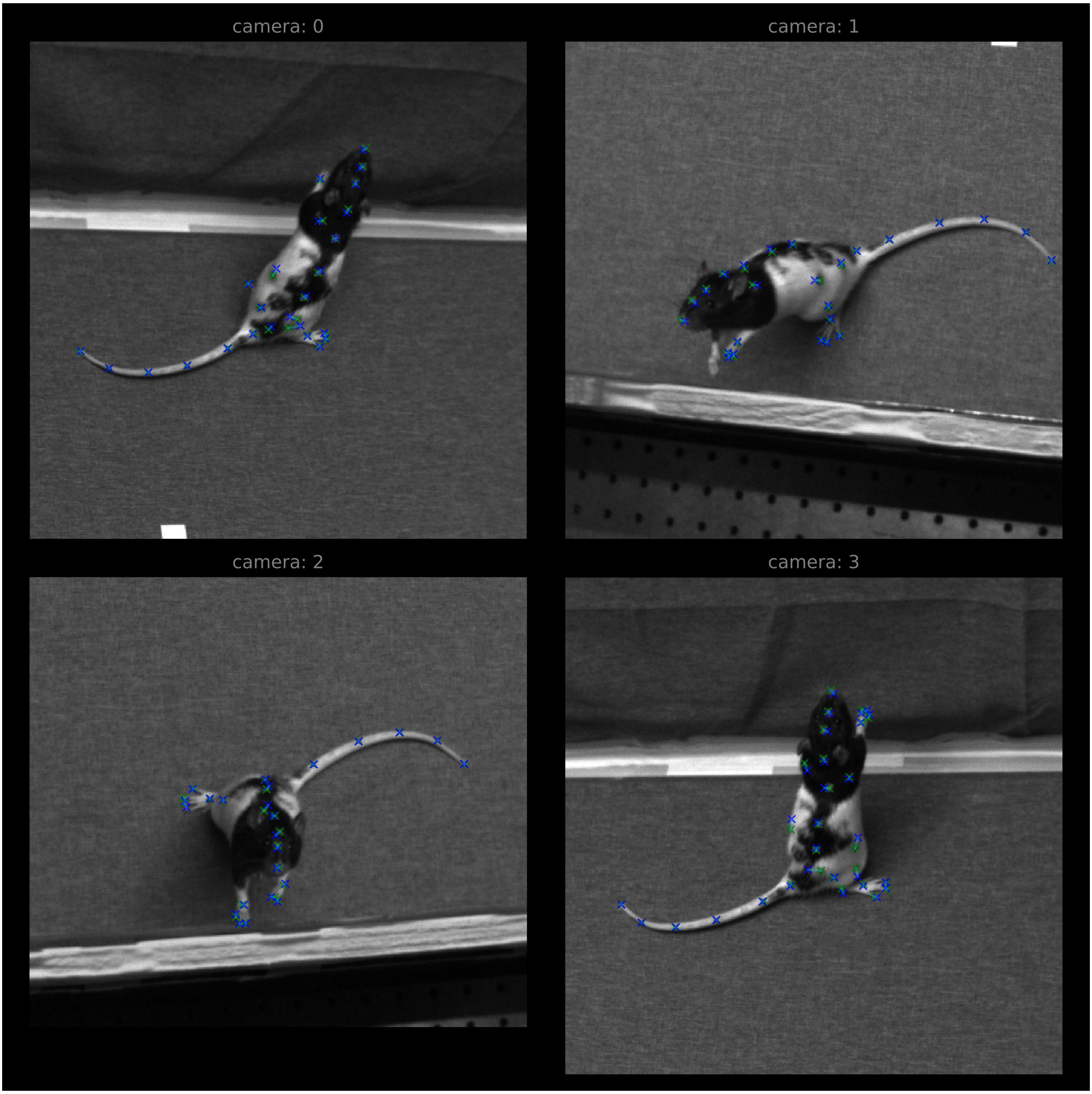

Supplementary Fig. 2 | Synchronous training frames from four calibrated cameras used for learning skeleton lengths and surface maker positions. The figure shows the manually labeled surface marker positions (green), their locations after the skeleton model is learned (blue) and the discrepancies between the two (green lines).

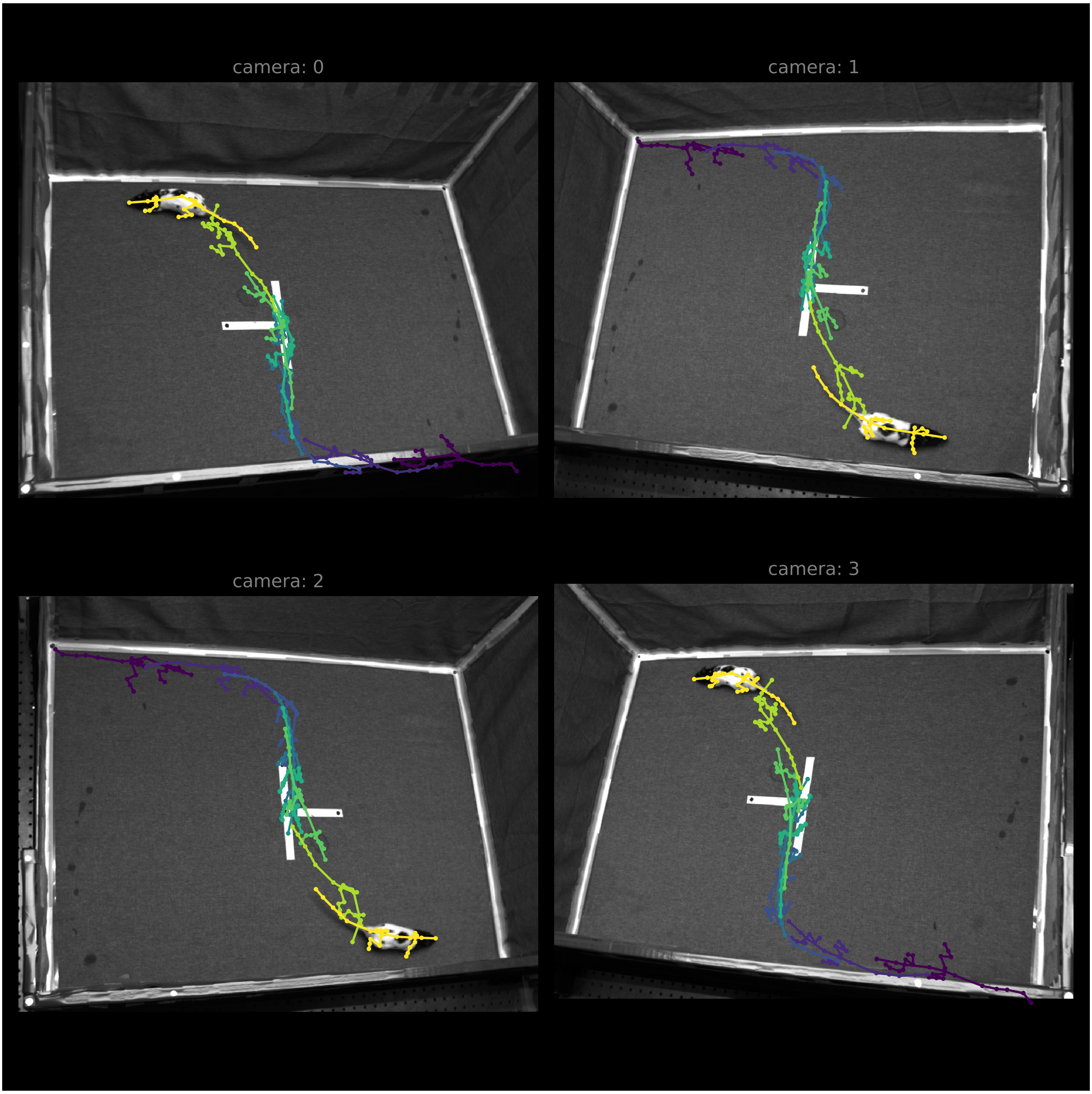

Supplementary Fig. 3 | Synchronous frames from four calibrated cameras which were part of the gait data set. Reconstructed skeleton poses are shown for different time points of the gait sequence. The time difference between poses is 1 s.

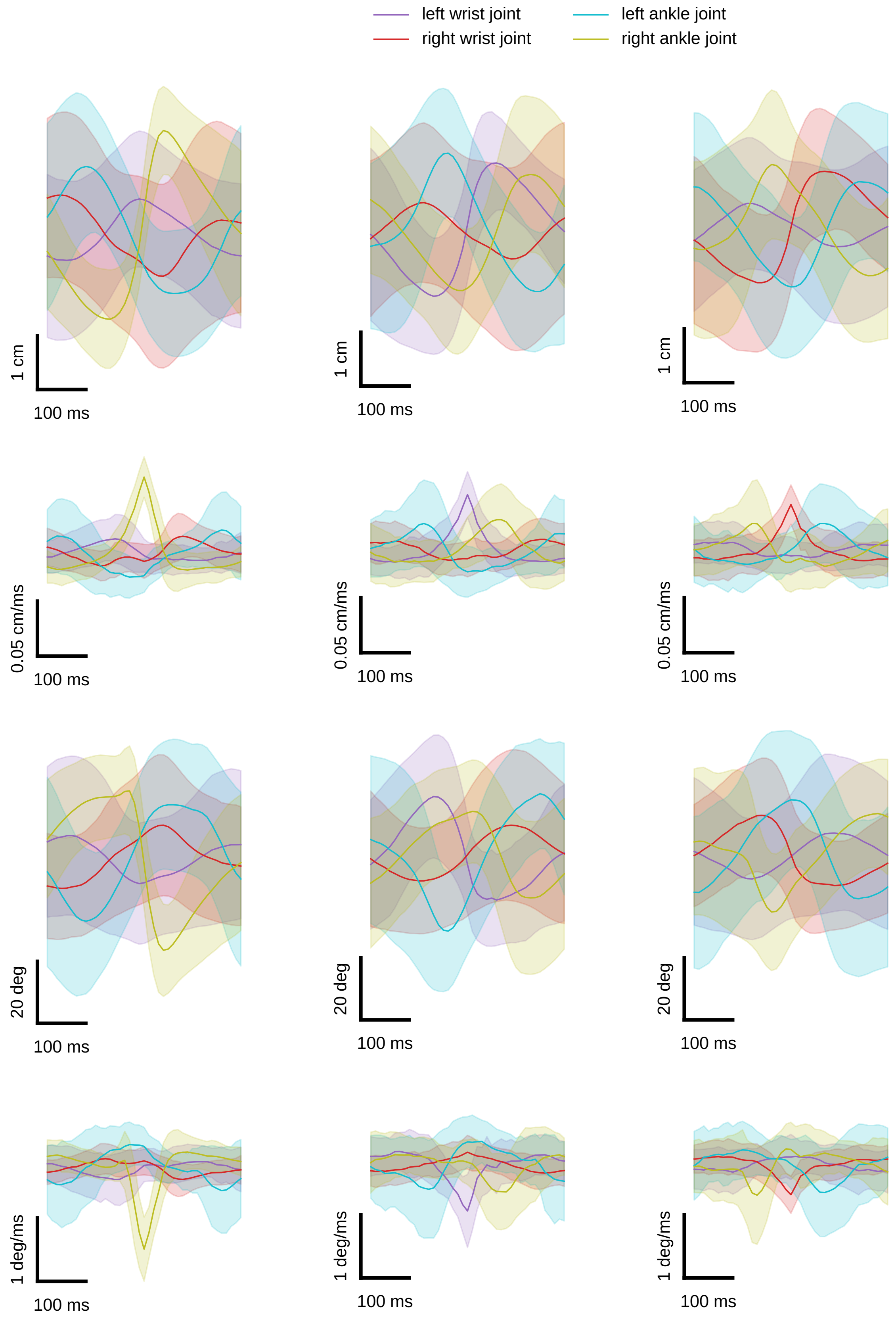

Supplementary Fig. 4 | Averaged traces from the ACM as in Figure 3d,g,j,m (left), but with trace alignment based on the velocity peaks for the right ankle (right column), left wrist (center column) and right wrist joint (right column), instead of aligning to the velocity peak of the left ankle joint.

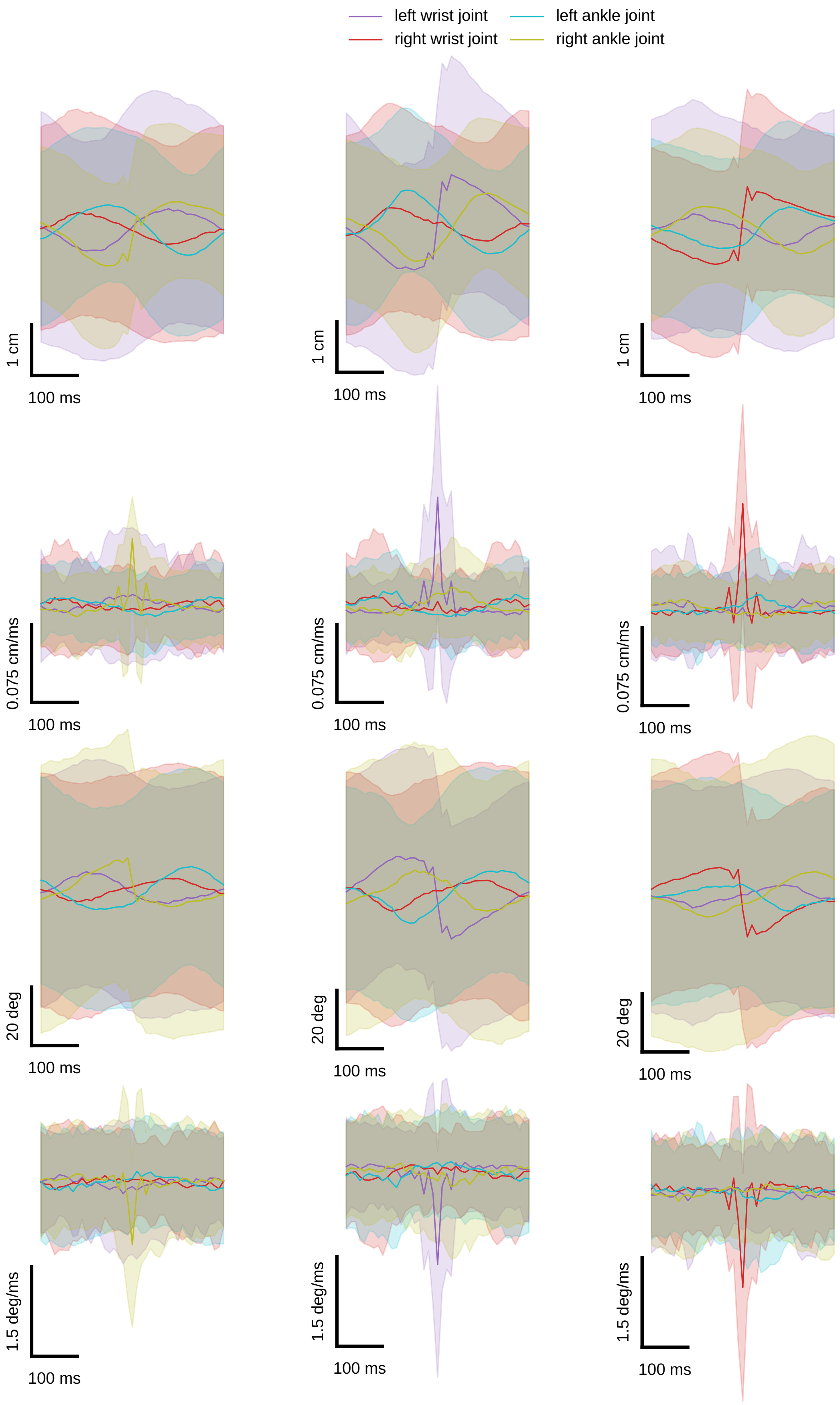

Supplementary Fig. 5 | Averaged traces from the model not constrained by either temporal or joint limit constraints as in Figure 3d,g,j,m (right) aligned to velocity peaks for the right ankle (right column), left wrist (center column) and right wrist joint (right column), instead of aligning to the velocity peak of the left ankle joint.

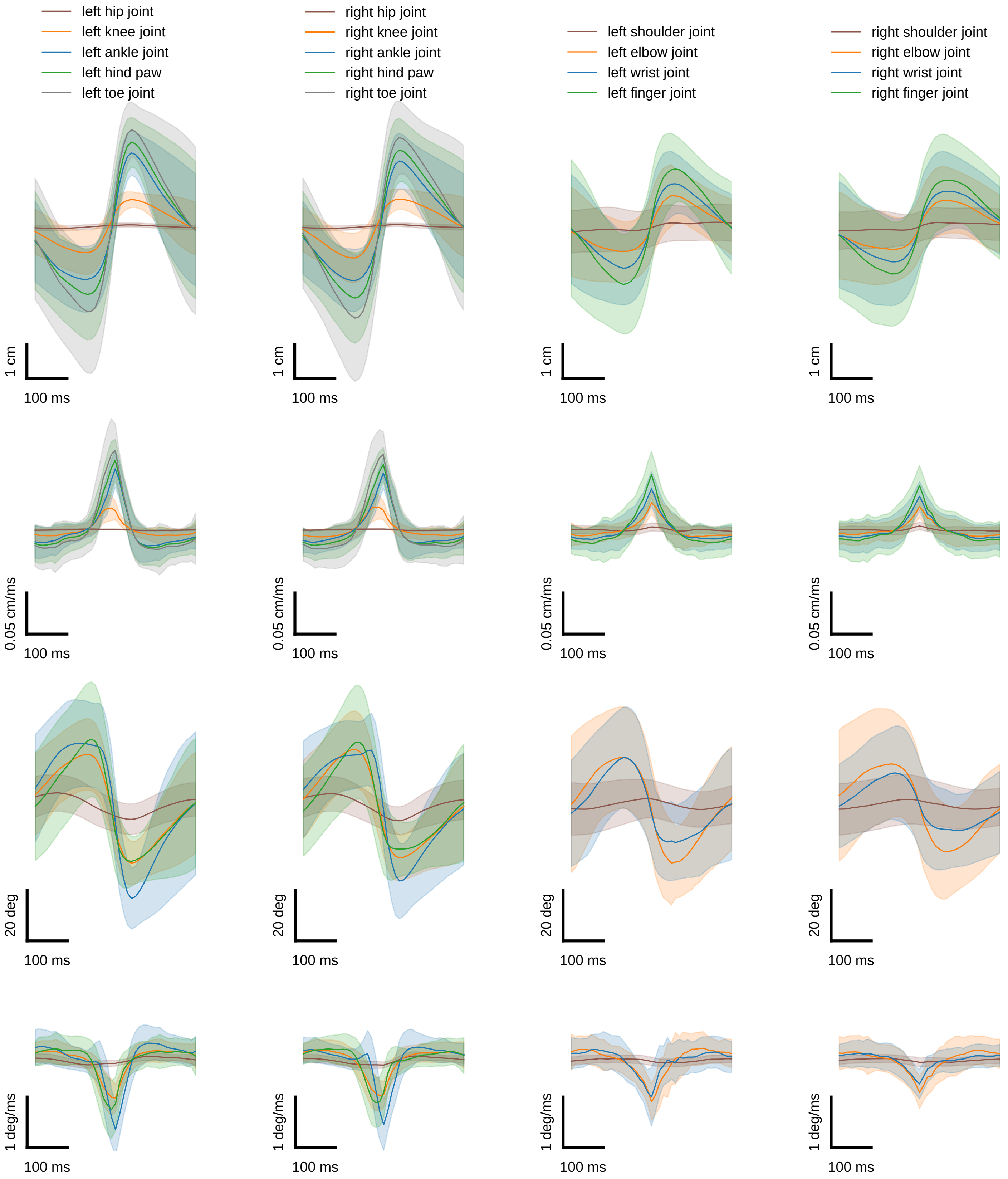

Supplementary Fig. 6 | Averaged traces from the ACM for all joints of the left hind (left column), right hind (center left column), left front (center right column) and right front limb (right column). Traces were aligned to velocity peaks from the left ankle, right ankle, left wrist and right wrist joint of the respective limb (mean±std of 1000 propagations through the probabilistic model).

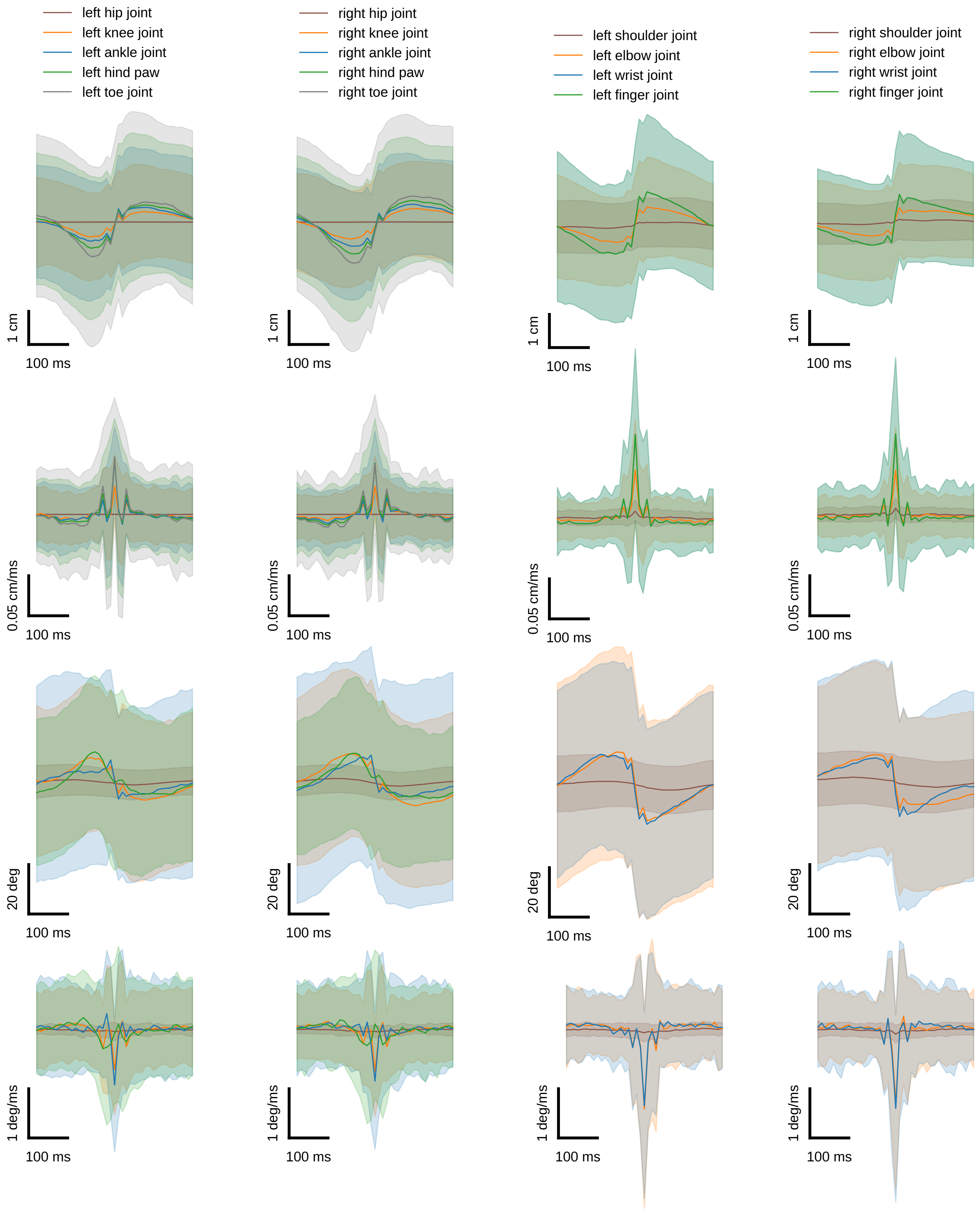

Supplementary Fig. 7 | Averaged traces from the model not constrained by either temporal or joint limit constraints for all joints of just the left hind (left column), right hind (center left column), left front (center right column) and right front limb (right column). Traces were aligned to velocity peaks from the left ankle, right ankle, left wrist and right wrist joint of the respective limb (mean±std of 1000 propagations through the probabilistic model).

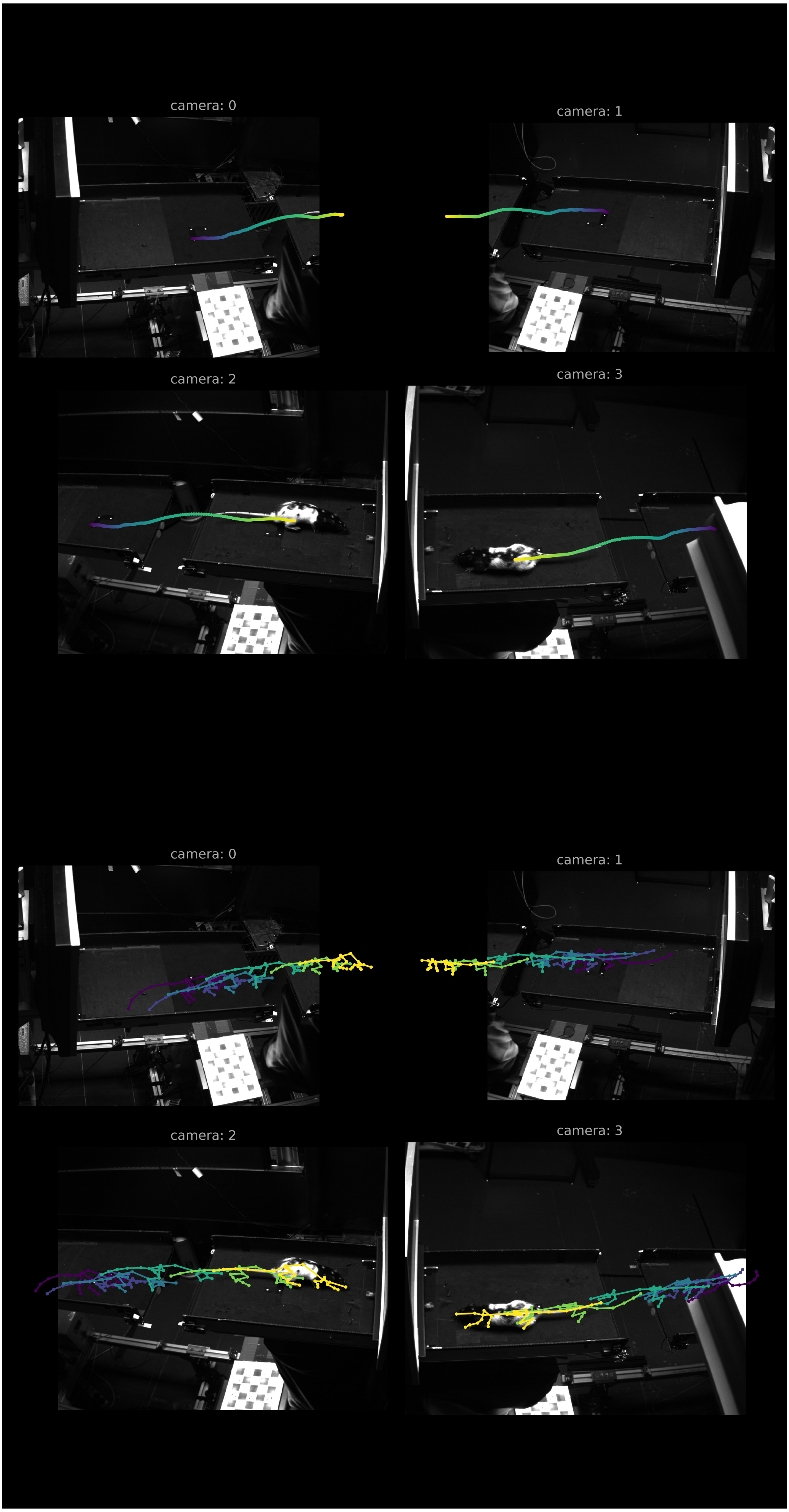

Supplementary Fig. 8 | Synchronous frames from four calibrated cameras which were part of the gap-crossing data set, with overlay of the center of mass (left) and reconstructed skeleton poses (right) shown for different time points (mean±std of 1000 propagations through the probabilistic model).

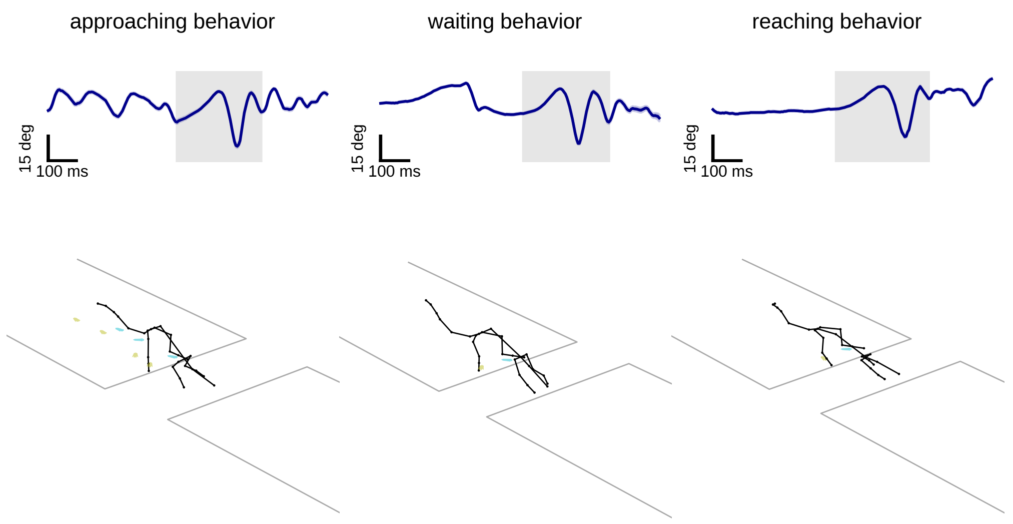

Supplementary Fig. 9 | Averaged joint-angle trace (average angles of all spine and hind paw joints) as a function of time (top) and reconstructed poses (bottom), showing hind paw footprints (cyan/yellow), at the start of a jump for three different gap crossing events. The animal was approaching the gap fast, with a smooth transition from walking to jumping behavior (left), waiting at the edge (center) or reaching to the other side of the track with its right front limb (right) before crossing the gap (mean±std of 1000 propagations through the probabilistic model).

State Space Model

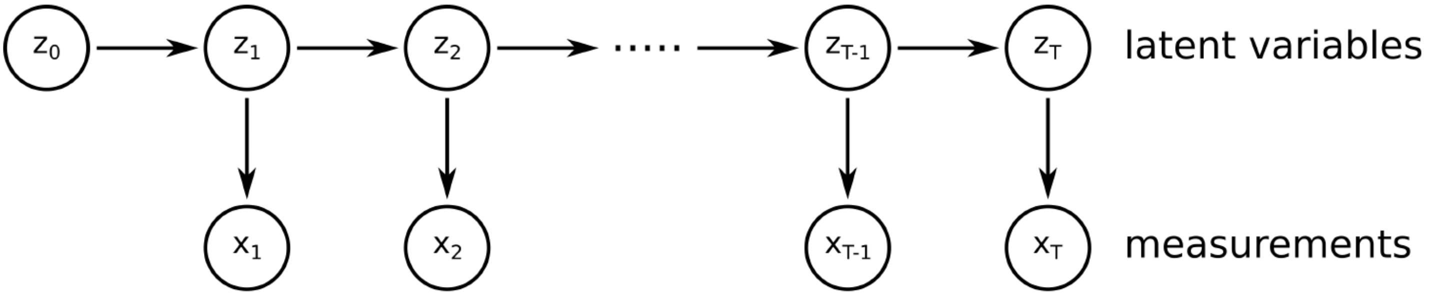

$$z_t = z_{t-1} + \epsilon_z$$
$$x_t = g(z_t) + \epsilon_x$$

$$z_0 \sim \mathcal{N}(\mu_0, V_0)$$
$$\epsilon_z \sim \mathcal{N}(0.0, V_z)$$
$$\epsilon_x \sim \mathcal{N}(0.0, V_x)$$

model parameters:

$$\theta = \{\mu_0, V_0, V_z, V_x\}$$

Supplementary Fig. 10 | Illustration of the state space model used for describing behavioral time series'.

Joint	x (°)	y (°)	z (°)
left shoulder	[25,205]	[-85,25]	[-35,35]
right shoulder	[25, 205]	[-25,85]	[-35,35]
left elbow	[2.5,145]	[0,0]	[-100,45]
right elbow	[2.5,145]	[0,0]	[-45,100]
left wrist	[-135,35]	[-12.5,37.5]	[0,0]
right wrist	[-135,35]	[-37.5,12.5]	[0,0]
left hip	[35,195]	[-65,25]	[-85,40]
right hip	[35,195]	[25,65]	[-40,85]
left knee	[-145,15]	[0,0]	[0,0]
right knee	[-145,15]	[0,0]	[0,0]
left ankle	[-10,145]	[0,0]	[0,0]
right ankle	[-10,145]	[0,0]	[0,0]
left metatarsophalangeal	[0,0]	[0,0]	[-15,35]
right metatarsophalangeal	[0,0]	[0,0]	[-35,15]

Supplementary Table 1. ACM joint angle limits.

Bone name	Rat<br>Slope avg. ± SD (cm/g)	Mouse<br>Length avg. ± SD (cm)
humerus	0.0075 ± 0.0005	1.162 ± 0.039
radius	0.0069 ± 0.0004	1.531 ± 0.046
metacarpal	0.0023 ± 0.0001	0.192 ± 0.009
femur	0.0102 ± 0.0006	1.668 ± 0.029
tibia	0.0114 ± 0.0006	1.809 ± 0.052
metatarsal	0.0053 ± 0.0003	0.639 ± 0.020

Supplementary Table 2. ACM Bone length constraints.

Start joint	End joint	Corresponding anatomical entity
joint_spine_002 (MRI: _003)	joint_hip_left	pelvic (half)
joint_hip_left	joint_knee_left	femur
joint_knee_left	joint_ankle_left	tibia
joint_ankle_left	joint_toe_left_002	pes
joint_shoulder_left	joint_elbow_left	humerus
joint_elbow_left	joint_wrist_left	radius
joint_spine_004 (MRI: _027)	joint_shoulder_left	clavicle

Supplementary Table 3. Joints used for ACM to MRI ground truth comparison.
