# Supplementary material for: Estimation of skeletal kinematics in freely moving rodents
Source: Nat Methods. 2022 Oct 17;19(11):1500–9. doi: 10.1038/s41592-022-01634-9 (PMC9636019; doi:10.1038/s41592-022-01634-9)
Supplement: Supplementary file 2 — Reporting Summary [file 41592_2022_1634_MOESM2_ESM.pdf]

nature research

Corresponding author(s): Jason Kerr

Last updated by author(s): Aug 30, 2022

Reporting Summary

Nature Research wishes to improve the reproducibility of the work that we publish. This form provides structure for consistency and transparency in reporting. For further information on Nature Research policies, see our Editorial Policies and the Editorial Policy Checklist.

Statistics

For all statistical analyses, confirm that the following items are present in the figure legend, table legend, main text, or Methods section.

n/a	Confirmed	
☐	☒	The exact sample size (*n*) for each experimental group/condition, given as a discrete number and unit of measurement
☐	☒	A statement on whether measurements were taken from distinct samples or whether the same sample was measured repeatedly
☐	☒	The statistical test(s) used AND whether they are one- or two-sided<br>*Only common tests should be described solely by name; describe more complex techniques in the Methods section.*
☒	☐	A description of all covariates tested
☐	☒	A description of any assumptions or corrections, such as tests of normality and adjustment for multiple comparisons
☐	☒	A full description of the statistical parameters including central tendency (e.g. means) or other basic estimates (e.g. regression coefficient) AND variation (e.g. standard deviation) or associated estimates of uncertainty (e.g. confidence intervals)
☐	☒	For null hypothesis testing, the test statistic (e.g. *F*, *t*, *r*) with confidence intervals, effect sizes, degrees of freedom and *P* value noted<br>*Give P values as exact values whenever suitable.*
☒	☐	For Bayesian analysis, information on the choice of priors and Markov chain Monte Carlo settings
☒	☐	For hierarchical and complex designs, identification of the appropriate level for tests and full reporting of outcomes
☐	☒	Estimates of effect sizes (e.g. Cohen's *d*, Pearson's *r*), indicating how they were calculated

Our web collection on statistics for biologists contains articles on many of the points above.

Software and code

Policy information about availability of computer code

Data collection	Data was acquired using custom-written MATLAB software for synchronously recording images from multiple Basler cameras. Cameras were synchronized using an external Teensy 3.6 microcontroller and custom-written Python software.
Data analysis	The ACM pipeline was written using Python 3.7.6, and the following packages: autograd 1.3, cudnn 7.6.5, numpy 1.18.1, jax 0.2.0, pytorch 1.4.0, scipy 1.4.1, tensorboard 1.14.0 and tensorflow 1.14.0.2. The code is available at https://github.com/bbo-lab/ACM, including a link to a test dataset, installation and running instructions.

For manuscripts utilizing custom algorithms or software that are central to the research but not yet described in published literature, software must be made available to editors and reviewers. We strongly encourage code deposition in a community repository (e.g. GitHub). See the Nature Research guidelines for submitting code & software for further information.

Data

Policy information about availability of data

All manuscripts must include a data availability statement. This statement should provide the following information, where applicable:

- Accession codes, unique identifiers, or web links for publicly available datasets
- A list of figures that have associated raw data
- A description of any restrictions on data availability

The data present in all figures as well as compressed video files are available in the related Dryad repository https://doi.org/10.5061/dryad.g4f4qrfsw under CC0 1.0 Universal (CC0 1.0) Public Domain Dedication.

Field-specific reporting

Please select the one below that is the best fit for your research. If you are not sure, read the appropriate sections before making your selection.

☒ Life sciences ☐ Behavioural & social sciences ☐ Ecological, evolutionary & environmental sciences

For a reference copy of the document with all sections, see nature.com/documents/nr-reporting-summary-flat.pdf

Life sciences study design

All studies must disclose on these points even when the disclosure is negative.

Sample size	As this manuscript is a description of a newly developed algorithm for skeletal pose reconstruction of freely-moving animals and demonstration of its function, we did not predetermine a sample size. Instead we have made multiple replicates of all experiments and provide in the manuscript direct descriptions of the central tendency and associated errors and ranges of the outputs of the algorithm.
Data exclusions	No data was excluded.
Replication	Sixteen different data sets were acquired from eight individual rats and two different data sets collected from 2 mice, which we deemed an appropriate number given the variability and size of the different data sets as well as ethical considerations on the number of subjects. A range of different rat subjects were selected to represent a range of different body sizes to illustrate the general applicability of the method and data sets from mice acquired to illustrate that the method is generalizable across species. All replicates were successful.
Randomization	Sixteen different data sets were acquired from eight individual rat subjects and two additional data sets acquired from two individual mouse subjects. Randomization was not considered necessary as the results of the algorithm were not influenced by operator bias.
Blinding	Distinct experimental groups were not part of the experimental design, and consequently blinding was not necessary.

Reporting for specific materials, systems and methods

We require information from authors about some types of materials, experimental systems and methods used in many studies. Here, indicate whether each material, system or method listed is relevant to your study. If you are not sure if a list item applies to your research, read the appropriate section before selecting a response.

Materials & experimental systems

n/a	Involved in the study	
☒	☐	Antibodies
☒	☐	Eukaryotic cell lines
☒	☐	Palaeontology and archaeology
☐	☒	Animals and other organisms
☒	☐	Human research participants
☒	☐	Clinical data
☒	☐	Dual use research of concern

Methods

n/a	Involved in the study	
☒	☐	ChIP-seq
☒	☐	Flow cytometry
☒	☐	MRI-based neuroimaging

Animals and other organisms

Policy information about studies involving animals; ARRIVE guidelines recommended for reporting animal research

Laboratory animals	Subjects were eight male Lister Hooded rats (Rattus norvegicus domestica) weighing between 71 and 735 g at the beginning of the experiment and four adult male C57BL/6 mice aged between 55 and 61 weeks and weighing between 27 and 36 g at the beginning of the experiment. Rats were maintained on a reversed 12 hour light cycle, mice on a normal 12 hour light cycle. Humidity was controlled to be greater than 45% for both rats and mice, and was checked daily.
Wild animals	This study did not involve wild animals.
Field-collected samples	This study did not involve field-collected samples.
Ethics oversight	Ethical oversight was by the Landesamt für Natur, Umwelt und Verbraucherschutz, North Rhine-Westphalia, Germany.

Note that full information on the approval of the study protocol must also be provided in the manuscript.
